# Electronic Health Self-Management Interventions for Patients With Chronic Kidney Disease: Systematic Review of Quantitative and Qualitative Evidence

**DOI:** 10.2196/12384

**Published:** 2019-11-05

**Authors:** Hongxia Shen, Rianne M J J van der Kleij, Paul J M van der Boog, Xinwei Chang, Niels H Chavannes

**Affiliations:** 1 Department of Public Health and Primary Care Leiden University Medical Centre Leiden Netherlands; 2 Department of Obstetrics and Gynaecology Erasmus Medical Center Rotterdam Netherlands; 3 Department of Nephrology Leiden University Medical Centre Leiden Netherlands; 4 Department of Surgery School of Nutrition and Translational Research in Metabolism Maastricht University Maastricht Netherlands

**Keywords:** eHealth, self-management, systematic review, chronic kidney disease

## Abstract

**Background:**

Chronic kidney disease (CKD) poses a major challenge to public health. In CKD patients, adequate disease self-management has been shown to improve both proximal and distal outcomes. Currently, electronic health (eHealth) interventions are increasingly used to optimize patients’ self-management skills.

**Objective:**

This study aimed to systematically review the existing evidence regarding the implementation and effectiveness of eHealth self-management interventions for patients with CKD.

**Methods:**

Following a search in 8 databases (up to November 2017), quantitative and qualitative data on process and effect outcomes were extracted from relevant studies. Quality was appraised using the Crowe Critical Appraisal Tool; narrative synthesis was performed to analyze the data extracted.

**Results:**

Of the 3307 articles retrieved, 24 (comprising 23 studies) were included in this review; of these, almost half were appraised to be of low to moderate quality. There was considerable heterogeneity in the types of interventions used and the outcomes measured. A total of 10 effect and 9 process outcome indicators were identified. The most frequently reported effect outcome indicators were specific laboratory tests and blood pressure (BP), whereas satisfaction was the most frequently reported process outcome indicator. Positive effects were found for proximal outcomes (eg, BP control and medication adherence), and mixed effects were found for more distal outcomes (eg, quality of life). High feasibility, usability, and acceptability of and satisfaction with eHealth self-management interventions were reported. The determinant ability of health care professionals to monitor and, if necessary, anticipate on patient measurements online was mostly cited to influence patients’ adherence to interventions.

**Conclusions:**

eHealth self-management interventions have the potential to improve disease management and health outcomes. To broaden the evidence base and facilitate intervention upscaling, more detailed descriptions and thorough analysis of the intervention components used are required. In addition, our review reveals that outcomes closely related to the scope and duration of the intervention implemented are most likely to be impacted. For instance, if a 4-week Web-based training to optimize disease management skills is implemented, the outcome perceived control would more likely be affected than kidney function. Although this seems obvious, most studies evaluate only distal outcomes and thereby fail to capture intervention effects that might contribute to long-term health improvement. We advise future researchers to carefully consider their choice of outcomes based on their sensitivity for change. In this way, we ensure that relevant effects are captured and legitimate conclusions are drawn.

## Introduction

### Background

Chronic kidney disease (CKD) is a major public health concern [1-3]. Globally, more than 70 million individuals are affected by CKD [4]. CKD is defined as kidney damage or a measured glomerular filtration rate (GFR) of ≤60 mL/min/1.73m^2^ for more than 3 months. CKD is classified into 5 stages based on GFR decline [5]. The level of kidney function deterioration has a direct relationship with an increase in morbidity and mortality [6], poorer patient outcomes [3], higher hospitalization rates [7], and substantial increase in health care expenditures [8]. Patients with CKD report a lower quality of life (QoL) [9] and may experience severe medical complications and cognitive dysfunction [10].

Disease self-management (hereafter referred to as *self-management*) is defined as “an individual’s ability to manage the symptoms, treatment, physical and psychosocial consequences, and lifestyle changes inherent to the life with a chronic condition” [11]. Adequate self-management is reported to improve patients’ health behaviors targeted by the intervention (ie, proximal outcomes) and also indirect outcomes, such as disease characteristics and progress (ie, distal outcomes) [12-14]. Although the potential benefits of self-management interventions are widely reported in the literature, extrapolating these results in day-to-day practice is difficult. Lack of efficacy in practice might be related to a suboptimal implementation of the self-management interventions [15,16]. Reported barriers were often related to intervention characteristics, such as lack of tailoring to the individual patient. Moreover, a lack of patient involvement in intervention design and insufficient care continuity and accessibility were reported to hamper implementations [17,18].

Electronic health (eHealth) technologies can help address implementation barriers by making interventions more accessible, acceptable, tailored, and interactive [19-21]. The most cited definition of eHealth is that by Eysenbach [22]:

e-health is [...] referring to health services and information delivered or enhanced through the Internet and related technologies. In a broader sense, characterizes [...] to improve health care locally, regionally, and worldwide by using information and communication technology.

eHealth can help patients achieve personal health goals, and it allows them to feel more responsible for their health status [23]. Moreover, eHealth facilitates remote patient communication and exchange of health data, helping to increase health care efficiency while maintaining a wide-scale, cost-effective health care approach [24]. eHealth interventions have been successfully implemented to support weight loss [25,26], promote smoking cessation [27], reduce depressive symptoms [28], and decrease mortality rates and acute admissions [29]. In addition, eHealth-based interventions have been successfully applied to manage chronic disease [30-32].

Several studies have reported the use of eHealth-based self-management interventions in CKD [33-36]. Moreover, 3 systematic reviews were published on this topic [37-39]. However, these reviews only concentrated on 1 particular eHealth application, such as telemedicine; dietary mobile apps; and automated information technology tools. Moreover, these reviews focused on a limited number of study designs and outcomes. For example, 2 reviews only included randomized controlled trials (RCTs) [38,39], and 1 review excluded studies focusing on implementation outcomes such as feasibility, validity, and acceptability [39]. Moreover, none of these reviews [37-39] reviewed the contribution of individual intervention components (eg, self-monitoring) to the effects found. These limitations of previous reviews make it difficult for researchers and intervention developers to determine which components should be employed to maximize the effectivity of eHealth self-management interventions for CKD patients.

### Objectives

This study, therefore, aimed to systematically review the available evidence on eHealth-based self-management interventions for CKD. In specific, we aimed to review the following: (1) study characteristics and type of eHealth applications used; (2) intervention components implemented and, if possible, their relative contribution to the effect found; (3) both process and effect outcomes; and (4) determinants of implementation.

## Methods

### Protocol and Registration

This review was performed according to the Preferred Reporting Items for Systematic Reviews and Meta-Analyses (PRISMA) statement [40]. The protocol was registered in the international Prospective Register of Systematic Reviews database (Centre for Reviews and Dissemination [CRD] number: CRD 420 180 81681).

### Search Methodology

A systematic search was conducted to identify relevant articles; the search strategy was developed in collaboration with a certified librarian. In total, 8 electronic databases (PubMed, EMBASE, Web of Science, Cochrane Library, EmCare, PsycINFO, Academic Search Premier, and Science Direct) were searched in November 2017. Search terms covered 3 areas: (1) CKD, (2) eHealth, and (3) self-management (see Multimedia Appendix 1). Reference lists of the included studies were searched to identify other relevant articles. EndNote X9 (Clarivate Analytics) was used to support the review process.

### Eligibility Criteria

Inclusion and exclusion criteria (Textbox 1) were determined using the Patients, Interventions, Comparison, Outcomes, Study design methodology [41].

**Inclusion and exclusion criteria for this study.**

Inclusion criteria:

- Participants—patients classified with chronic kidney disease (stage 1-5)
- Intervention—eHealth technologies (“any information and communication technology designed to deliver or enhance health services and information”) applied to facilitate chronic kidney disease patients’ self-management (“the care taken by individuals towards their own health and well-being: it comprises the actions they take to lead a healthy lifestyle; to meet their social, emotional and psychological needs; to care for their long-term condition, and to prevent further illness or accidents”) [11]
- Comparison—no restrictions
- Outcomes—articles reporting on clinical (ie, patients’ intermediate outcomes or clinical parameters of disease severity, such as blood pressure, fluid management, and mortality), humanistic (ie, consequences of disease or treatment on patients’ functional status or quality of life, such as physical functioning, well-being, and levels of depression or anxiety), economic and utilization (ie, measures of health resource utilization, medical costs, and cost-effectiveness), and/or process (ie, indicators that affect patient care by improving health care delivery or patient-health care interactions and self-management related–factors, such as adherence to intervention, usability of eHealth technologies, and self-efficacy) outcomes
- Language restrictions—articles needed to be written in English
- Study design—randomized and nonrandomized controlled trials, noncomparative trials, and qualitative or mixed methods articles

Exclusion criteria:

- Type of electronic health used—studies with devices only used for communication (eg, a telephone only used for a follow-up call) or data collection (eg, an internet system solely used to collect patient data without further intervention) purposes
- Study design—case reports containing ≤3 participants, commentaries, reviews, letters, dissertations, editorials, conference proceeding, and books

### Study Identification

After removal of duplications, titles and abstracts of the retrieved articles were screened independently by 2 reviewers (HS and XC). Articles that did not meet inclusion criteria were removed. Potentially relevant articles were obtained in full text and reviewed independently by 2 authors (HS and XC). Any disagreements between the 2 authors were resolved by consensus or consultation with a third author (RK).

### Data Collection

Data collection was performed independently by 2 reviewers (HS and XC) using a standardized data extraction form. Study characteristics, descriptions of eHealth self-management interventions (eg, intervention components), process and effect outcome indicators, and determinants of implementation were extracted. Discrepancies in extraction were discussed until consensus was reached.

### Quality Assessment

Article quality was appraised independently by HS and XC using the Crowe Critical Appraisal Tool (CCAT) [42]—a reliable, widely used quality appraisal tool [43,44]. Use of the CCAT user guide promoted validity and inter-rater reliability [43-46]. The CCAT form is divided into 8 categories and 22 items, with a total of 99 subitems. Subitems are rated on a scale of *present*, *absent*, or *not applicable*. A 6-point scale ranging from 0 (the lowest) to 5 (the highest) is used to assign score per category, with 40 being the maximum achievable total score.

The CCAT does not allow for a qualitative comparison of appraisal scores. Hence, we used the star score system developed by our research group to compare study quality [47]. First, we calculated a quality score based on the CCAT. Then, a mean score and standard deviation of the quality scores were calculated. Star scores were then assigned to each article: 1 star if a quality score was more than 1 SD below mean; 2 stars if a quality score ranged from 1 SD below mean to mean score. The kappa between the 2 reviewers’ scores of quality assessment was 0.63, reflecting substantial agreement [48].

### Data Synthesis

Data were reviewed using narrative synthesis [49]. Study characteristics were reviewed, summarized, and analyzed in a spreadsheet. In accordance with previous categorizations of eHealth [32,39,50], eHealth self-management interventions were split into 5 major types (see Multimedia Appendix 2). eHealth functionalities used were described based on the technology functionality framework [51,52]. In addition, based on the operationalization by Mohr et al [53], eHealth-based self-management interventions included were further detailed: (1) intervention components (based on Morrison et al [54]; see Multimedia Appendix 3)—active intervention parts that support self-management behavior, including elements defined as *what* is provided to the user (eg, education materials, integrated alerts, and video conferencing options), *how* these elements are delivered (eg, plans and quizzes), and the subsequent intervention workflow defined as *when* they are delivered (eg, daily use)—and (2) intervention strategies—behavior change techniques [55] that underlie the intervention components (eg, *role modeling* if the Web-based education materials used include a video of patient who successfully manages his/her disease).

Outcome indicators were classified into 2 categories: effect outcome indicators and process outcome indicators [56]. Effect outcome indicators were outcomes related to self-management, health status, or cost-effectiveness, whereas process outcome indicators were outcomes on care process, health care delivery, or patient-health care interactions (eg, adherence and usability).

To allow for comparability, we classified the results reported as *positive effect*, *no statistically significant effect*, or *mixed effect* (see Textbox 2). No negative outcomes were reported in the studies included in this review. Only quantitative methods were used to measure effect outcome indicators, whereas mixed methods were used to measure some process outcome indicators. Hence, the classification of the results of the process outcome indicators slightly differs from that of the effect outcome indicators. Outcomes related to patients and care providers are reported separately.

The determinants of implementation of eHealth self-management interventions extracted were categorized following the widely cited framework by Fleuren et al [57]. This framework identifies 50 determinants of program implementation in 5 subgroups: (1) characteristics of the sociopolitical context, such as legislation; (2) characteristics of the organization, such as staff turnover; (3) characteristics of the person adopting the innovations (user of the innovation), such as knowledge; (4) characteristics of the innovation, such as complexity; and (5) innovation strategies, such as a training. For example, the study by McGillicuddy et al [36] included in our review mentioned that “six subjects did not complete the lead-in phase, 5 for technical reasons relating to poor internet at their home.” This barrier was then mirrored to the 50 determinants in Fleuren framework and classified as a determinant related to the *innovation* and, more specifically, added to the determinant category *perceived quality of eHealth intervention is excellent*. In addition, in each subgroup, we identified the influence of the patients or care providers.

**Outcome indicators for electronic health self-management interventions.**

- Effect outcome indicators
  *Positive effect*—if, after statistical analysis, a significant effect was reported
  *No statistically significant effect*—if, after statistical analysis, a nonstatistically significant effect was reported or if no statistical analysis was performed
  *Mixed effect*—if results that could be classified as both positive and no effect were reported
- Process outcome indicators
  - *Positive effect—*if, after statistical analysis, a statistically significant effect was reported or if a positive effect or an improvement between certain points in time was reported (eg, interviews revealed that patients were highly satisfied with the electronic health application)
  - *No statistically significant effect—*if, after statistical analysis, a nonsignificant effect was reported or if a no effect or no differences between certain points in time was reported
  - *Mixed effect—*if results that could be classified as both positive and no effect were reported

## Results

### Study Selection

**Figure 1:**
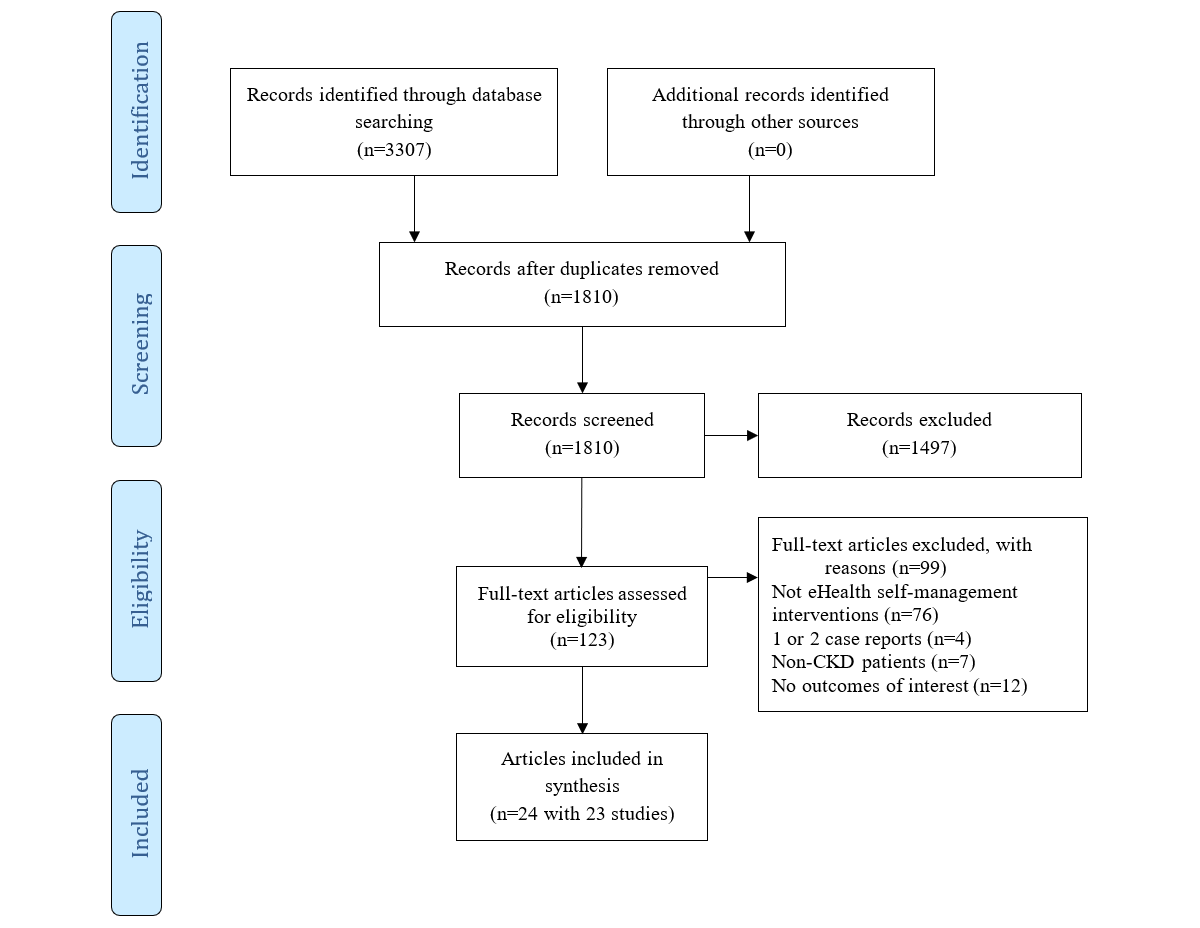
Preferred Reporting Items for Systematic Reviews and Meta-Analyses flowchart of the systematic review. CKD: Chronic kidney disease; eHealth: electronic health.

Our search retrieved 3307 articles in total. After removing 1497 duplicates, 1810 relevant articles were screened based on title and abstract. A total of 123 potentially relevant articles were screened full text. Of these papers, 2 described results of the same RCT [58,59] and were assessed jointly. Finally, 24 articles (comprising 23 studies) [33-36,58-77] were found eligible for inclusion in this review (Figure 1).

### Study Characteristics

All 23 studies were published between 2005 and 2017, with 19 of them being conducted between 2012 and 2017 [33-36,58,64-77]. A total of 13 studies were conducted in the United States [33-36,58,60,62-65,69,71,72], followed by 2 studies in the United Kingdom [70,74]. The research designs used varied; the majority used an RCT design [33-36,58,63,64,66,70]. Most studies focused on the usability, acceptability, and feasibility of eHealth self-management interventions [36,58,61,64,65,67,69,71,72,74-77]. Most participants are patients receiving hemodialysis [58,60,62-64,66,68,69,76,77]. Sample size at baseline ranged from 5 [67] to 601 [34]. Target population age ranged from 21 to 93 years. Recruitment mostly occurred via medical centers/hospitals [35,58,60-62,68,73]. Intervention duration ranged from 2 weeks [76] to 24 months [61]; 2 studies did not specify intervention duration [58,67]. A total of 10 studies performed a follow-up measurement [33,34,63,65,66,69-71,73,76]. Moreover, 12 studies included a control group, and 9 of those studies [33,34,36,58,61,66,68,70,76] reported *usual care* or no internet-delivered intervention as control condition. The study characteristics have been presented in Multimedia Appendix 4.

### Quality Appraisal Scores of Studies

Quality of the included articles varied (Table 1). A total of 3 articles [70,73,75] were awarded a 4-star rating, 11 [33-36,59,63,65,66,69,74,76] a 3-star rating, and 10 [58,60-62,64,67,68,71,72,77] a 2-star rating or lower. Articles with a 4-star rating scored higher on design, sampling, data collection, and ethics compared with those with a 3-star rating or lower. Moreover, 20 articles [34-36,59-70,72,73,75-77] provided insufficient details on their study design or rationale. Sampling method used (eg, randomly and purposively) was not reported in 10 articles [35,60,62,65,67,71,73,74,76,77]. Although both the number and characteristics of participants were described in most articles, 15 articles [58-62,64-69,71,74,76,77] did not specify the method of sample size calculation. A total of 10 articles [58-62,64,65,67,71,72] did not detail methods used to ensure the quality of the data collected or to reduce bias. On average, the lowest score was obtained on the *ethics* section.

**Table 1:** Quality appraisal scores on the Crowe Critical Appraisal Tool.

Study	Total score (maximum=40)	Star score^a^	Preamble	Introduction	Design	Sample	Data	Ethic	Result	Discussion
van Lint et al (2015) [73]	33	4-star	4	4	4	4	4	4	4	5
Blakeman et al (2014) [70]	32	4-star	5	4	4	5	3	3	4	4
Ong et al (2016) [75]	32	4-star	4	4	4	4	4	4	4	4
Forni Ogna et al (2013) [66]	31	3-star	5	4	3	4	3	4	4	4
Ishani et al (2016) [34]	31	3-star	4	4	4	4	3	3	4	4
Stark et al (2011) [63]	30	3-star	4	4	4	4	3	3	4	4
McGillicuddy et al (2013) [36]	30	3-star	4	4	4	4	3	3	4	4
Hayashi et al (2017) [76]	30	3-star	5	4	3	3	4	3	4	4
Diamantidis et al (2013) [65]	29	3-star	5	4	4	3	3	4	3	3
Reese et al (2017) [35]	29	3-star	5	5	4	3	2	2	3	5
Dey et al (2016) [74]	28	3-star	3	4	3	3	3	4	4	4
Berman et al (2011) [59]	27	3-star	5	4	3	3	2	3	4	3
Rifkin et al (2013) [33]	27	3-star	5	4	3	4	3	2	3	3
Welch et al (2013) [69]	27	3-star	3	4	4	3	3	3	4	3
Connelly et al (2012) [64]	26	2-star	5	5	3	4	3	1	2	3
Neumann et al (2013) [68]	26	2-star	3	4	3	3	3	4	3	3
Liu et al (2017) [77]	25	2-star	4	5	3	2	3	1	3	4
Diamantidis et al (2015) [72]	24	2-star	3	4	3	4	1	3	3	3
Minatodani et al (2013) [58]	23	2-star	3	4	2	1	3	4	3	3
Sevick et al (2005) [60]	22	2-star	3	4	3	2	2	2	3	3
Harrington et al (2014) [71]	20	1-star	3	4	2	3	3	0	3	2
Gallar et al (2007) [61]	18	1-star	2	2	3	2	1	3	2	3
Heiden et al (2013) [67]	18	1-star	3	4	3	1	1	0	3	3
Whitten et al (2008) [62]	14	1-star	2	4	1	1	1	0	1	4

^a^1-star: more than 1 SD below mean; 2-star, between 1 SD below mean and mean; 3-star, between mean and 1 SD above mean; 4-star, more than 1 SD above mean.

### Description of Electronic Health Self-Management Interventions

Major types of eHealth, functionalities, and key intervention components used are summarized in Tables 2 and 3. Most eHealth interventions evaluated included *multiple components* (multiple eHealth types) to improve patients’ self-management (8/23 articles). Studies included did not provide detail on the specific intervention strategies underpinning these components, such as behavior change techniques. The most frequently used intervention component was self-monitoring (17/23 articles), followed by educational material or training (15/23 articles) and counseling (14/23 articles). Less frequently used intervention components were quizzes (3/23 articles) and interactive feedback from a device (4/23 articles). In addition, 5 studies reported that intervention development was guided by a specific theory.

**Table 2:** Descriptions of electronic health for each report included in the review.

Category of eHealth^a^		Detailed eHealth	Functionality
**Personal digital assistant (references)**			
	Sevick et al (2005) [60]	Dietary self-Monitoring: meals logs	Record
	Stark et al (2011) [63]	Dietary self-Monitoring: meals logs	Record
	Connelly et al (2012) [64]	Dietary intake monitoring: self-monitor diet and feedback	Record
	Forni Ogna et al (2013) [66]	Electronic medication event monitoring: monitor adherence	Record; communicate
	Welch et al (2013) [69]	Dietary intake monitoring: self-monitor diet and feedback	Display; record
	Diamantidis et al (2015) [72]	Medication inquiry system: identifying the safety of medications with impaired renal function	Record; display; alert
**Telemedicine (references)**			
	Gallar et al (2007) [61]	Videoconferencing: connecting home to hospital	Communicate
	Whitten et al (2008) [62]	Videoconferencing: connecting clinics and health system	Communicate; education
**Computer (references)**			
	Harrington et al (2014) [71]	Tablet computer: recording data and reviewing medical findings	Display; record; communicate; alert
	Ishani et al (2016) [34]	Touch screen computer with peripherals	Record; communicate
	Heiden et al (2013) [67]	Educational tool, food analyzer database and diet registration, and decision support to binder dosage	Communicate; education; record
**Multiple components** **(references)**			
	Diamantidis et al (2013) [65]	Alert accessories linked to website/safe kidney care: offering information	Record; education
	McGillicuddy et al (2013) [36]	BP^b^ monitoring, electronic medication tray, and mobile phone	Alert; communicate
	Minatodani et al (2013) [58], Berman et al (2011) [59]	Self-monitoring devices	Record; communicate
	Blakeman et al (2014) [70]	Website: tailoring access to community resources	Display; communicate
	Dey et al (2016) [74]	Computer tablet, wearable devices, and Web portal	Record; alert
	Ong et al (2016) [75]	Smartphone, a Web-based dashboard application, and a data server	Record; alert; display
	Hayashi et al (2017) [76]	Self-management and recording system for dialysis (wearable devices, smartphone, and administrator module)	Record; alert; display
	Liu et al (2017) [77]	App installed on mobile, cloud server, and Web app	Record; alert; communicate
**Wearable devices** **(references)**			
	Neumann et al (2013) [68]	Telemetric weight monitoring	Display; alert
	Rifkin et al (2013) [33]	BP monitoring	Record
	van Lint et al (2015) [73]	BP monitoring and creatine monitoring	Record
	Reese et al (2017) [35]	Wireless pill bottle	Record; alert

^a^eHealth: electronic health.

^b^BP: blood pressure.

**Table 3:** Descriptions of electronic health self-management interventions for each report included in the review.

Category of electronic health		Intervention components										Theory-based
		Educational material or training	Plan/ goals	Self- monitoring	Interactive feedback from device	Message/ alert to health caregivers	Message/ alerts to patients from device	Message/ alert to patients from health caregivers	Quizzes	Counseling	Daily use	
**Personal digital assistant (references)**												
	Sevick et al (2005) [60]	✓	✓	✓	—^a^	—	—	✓	—	✓	✓	✓
	Stark et al (2011) [63]	✓	✓	✓	—	—	—	✓	—	✓	✓	✓
	Connelly et al (2012) [64]	✓	—	✓	✓	—	—	—	—	✓	—	✓
	Forni Ogna et al (2013) [66]	—	✓	—	—	—	—	—	—	✓	—	—
	Welch et al (2013) [69]	✓	—	✓	—	—	—	—	—	✓	—	✓
	Diamantidis et al (2015) [72]	✓	—	—	✓	—	✓	—	—	—	—	—
	Total (N=6), n (%)	5 (83)	3 (50)	4 (67)	2 (33)	0	1 (17)	2 (33)	0	5 (83)	2 (33)	4 (67)
**Telemedicine (references)**												
	Gallar et al (2007) [61]	—	—	—	—	—	—	—	—	✓	—	—
	Whitten et al (2008) [62]	—	—	—	—	—	—	—	—	✓	—	—
	Total (N=2), n (%)	0 (0)	0 (0)	0 (0)	0 (0)	0 (0)	0 (0)	0 (0)	0 (0)	2 (100)	0 (0)	0 (0)
**Computer (references)**												
	Harrington et al (2014) [71]	—	✓	✓	—	✓	—	✓	—	—	✓	—
	Ishani et al (2016) [34]	✓	✓	✓	—	—	—	✓	—	—	—	—
	Heiden et al (2013) [67]	—	—	✓	—	—	—	—	—	—	—	—
	Total (N=3), n (%)	1 (33)	2 (67)	3 (100)	0 (0)	1 (33)	0 (0)	2 (67)	0 (0)	0 (0)	1 (33)	0 (0)
**Multiple components (references)**												
	Diamantidis et al (2013) [65]	✓	—	—	—	—	—	—	—	—	—	—
	McGillicuddy et al (2013) [36]	✓	✓	✓	—	✓	✓	✓	—	✓	✓	✓
	Minatodani et al (2013) [58], Berman et al (2011) [59]	✓	✓	✓	—	—	—	✓	✓	✓	—	—
	Blakeman et al (2014) [70]	✓	—	—	—	—	—	—	—	✓	—	—
	Dey et al (2016) [74]	✓	—	✓	—	✓	—	✓	✓	✓	—	—
	Ong et al (2016) [75]	✓	✓	✓	✓	✓	✓	—	—	—	—	—
	Hayashi et al (2017) [76]	—	✓	✓	—	—	✓	✓	—	✓	✓	—
	Liu et al (2017) [77]	—	—	✓	—	—	✓	—	✓	—	—	—
	Total (N=8), n (%)	6 (75)	4 (50)	6 (75)	1 (13)	3 (38)	4 (50)	4 (50)	3 (38)	5 (63)	2 (25)	1 (13)
**Wearable devices (references)**												
	Neumann et al (2013) [68]	—	✓	✓	—	✓	—	✓	—	✓	✓	—
	Rifkin et al (2013) [33]	✓	—	✓	—	—	—	✓	—	✓	—	—
	van Lint et al (2015) [73]	✓	✓	✓	—	—	—	—	—	—	—	—
	Reese et al (2017) [35]	✓	✓	✓	✓	✓	✓	✓	—	—	—	—
	Total (N=4), n (%)	3 (75)	3 (75)	4 (100)	1 (25)	2 (50)	1 (25)	3 (75)	0 (0)	2 (50)	1 (25)	0 (0)

^a^Not applicable.

### Summary of Results

Tables 4 and 5 present the outcome indicators and the data collection tools used. Moreover, full details on the efficacy data reported in the included studies are included in Multimedia Appendix 5. Table 6 displays the determinants of implementation extracted. No articles reported any adverse outcomes of eHealth self-management interventions.

**Table 4:** Summary of outcome indicators of electronic health self-management interventions.

Outcome category and indicator		Total number of articles in each category	Effect and references		
			Positive, n (%)	No statistically significant effect, n (%)	Mixed, n (%)
**Patient effect outcome (N=33)**					
	Blood pressure	5	4 (80) [36,68,70,75]^a^	1 (20) [33]	0 (0)
	Quality of life	4	1 (25) [70]^a^	2 (50) [59,74]	1 (25) [76]
	Laboratory tests	6	2 (33) [66,68]^a^	4 (67) [60,62,75,76]	0 (0)
	Interdialytic weight gain	4	1 (25) [68]^a^	3 (75) [60,69,76]	0 (0)
	Morbidity and mortality	2	0 (0)	2 (100) [34,61]	0 (0)
	Hospitalization rate and emergency room visit	3	2 (67) [61,59]^a^	1 (33) [34]	0 (0)
	Medical cost	2	1 (50) [59]^a^	1 (50) [61]	0 (0)
	Cost-effectiveness	1	1 (100) [70]^a^	0 (0)	0 (0)
	Nutrition and dietary intake	2	0 (0)	2 (100) [62,69]	0 (0)
	Medication adherence	4	3 (75) [35,36,66]^a^	1 (25) [33]	0 (0)
**Process outcome (N=28)**					
	Acceptability	6	6 (100); [69,74,76]^b^; [33,36,75]^c^	0 (0)	0 (0)
	Usability	5	5 (100); [64,67,76]^b^; [62,77]^c^	0 (0)	0 (0)
	Satisfaction	8	8 (100); [36,58,71-74,76]^b^; [75]^c^	0 (0)	0 (0)
	Adherence to intervention	4	4(100);[35,63,73,75]^b^	0 (0)	0 (0)
	First entry and length of dwell time	1	1 (100); [65]^b^	0 (0)	0 (0)
	Self-efficacy	1	0 (0)	1 (100); [69]^b^	0 (0)
	Perceived benefits	1	0 (0)	1 (100); [69]^b^	0 (0)
	Perceived control	1	1 (100); [69]^a,^^b^	0 (0)	0 (0)
	Recorded errors	1	1 (100); [72]^b^	0 (0)	0 (0)

^a^Statistically significant.

^b^Outcome related to patient.

^c^Outcome related to both patient and care provider.

**Table 5:** Summary of reported tools of outcome indicators.

Outcome category and indicator		Reported data collection tools (number of articles)
**Patient effect outcome (N=33), all quantitative**		
	Blood pressure	Readings (4) and dataset (1)
	Quality of life	36-item Short Form Health Survey (1), EuroQoL-5 Dimension (1), and 36-item Kidney Disease Quality of Life survey (2)
	Laboratory tests	Medical records (6)
	Interdialytic weight gain	Medical records (4)
	Morbidity and mortality	Charlson comorbidity index (1) and records (1)
	Hospitalization rate and emergency room visit	Records (3)
	Medical cost	Records (2)
	Cost-effectiveness	Records (1)
	Nutrition and dietary intake	Clinical data (2)
	Medication adherence	System data (2), adherence score calculation (1), and Morisky Medication Adherence Scale (1)
**Process outcome (N=28)**		
	Acceptability	Quantitative: questionnaires (1), recruitments and participation rate (1), QUEST^a^ and retention rates (1), and average number of daily entries and completion rates (2); quantitative and qualitative: number of assessments and semistructured interview (1)
	Usability	Quantitative: survey (1) and questionnaire (2); qualitative: interview (1); quantitative and qualitative: survey, interview, and system data (1)
	Satisfaction	Quantitative: questionnaires and QUEST (5); qualitative: semistructured interview (2); quantitative and qualitative: questionnaire and interview (1)
	Adherence to intervention	Quantitative: system data (3) and Basel Assessment of Adherence to Immunosuppressive Medications Scale (1)
	First entry and length of dwell time	Quantitative: frequency and number (1)
	Self-efficacy	Quantitative: cardiac diet self-efficacy and Fluid Self-Efficacy Scale (1)
	Perceived benefits	Quantitative: Benefits of Sodium Adherence and a 9-item Benefits of Fluid Adherence Scale (1)
	Perceived control	Quantitative: 7-item Mastery scale (1)
	Recorded errors	Quantitative: questionnaire and record (1)

^a^QUEST: Quebec user evaluation of satisfaction with assistive technology.

**Table 6:** Determinants of the implementation of electronic health self-management interventions for chronic kidney disease.

Determinants of interventions and details			References	
			If determinant is present	If determinant is exact opposite
**Sociopolitical context (patient)**				
	Awareness of potential health benefits of the eHealth^a^ self-management intervention		Berman et al (2011) [59], Hayashi et al (2017) [76]	—^b^
	Target population feels comfortable about eHealth use		Hayashi et al (2017) [76], Liu et al (2017) [77]	—
**Organization (patient)**				
	Community resources (eg, activities, services, and applicable wireless fidelity connection at the users’ location) available for implementation		Blakeman et al (2014) [70]	Harrington et al (2014) [71]
**User**				
	**Patient**			
		Support from colleagues (eg, internet personnel)	Stark et al (2011) [63]	McGillicuddy et al (2013) [36]
		Ability of health care professionals to monitor and, if necessary, anticipate on patient measurements online	Rifkin et al (2013) [33], Reese et al (2017) [35], Berman et al (2011) [59], van Lint et al (2015) [73], Ong et al (2016) [75], Liu et al (2017) [77]	—
		Availability of sufficient skills/knowledge	Diamantidis et al (2015) [72]	Berman et al (2011) [59], Welch et al (2013) [69]
		eHealth technology is considered valuable by user	McGillicuddy et al (2013) [36], Heiden et al (2013) [67], Blakeman et al (2014) [70]	van Lint et al (2015) [73]
		High self-efficacy	van Lint et al (2015) [73]	—
	**Patient and care provider**			
		eHealth technology is considered valuable by user	Rifkin et al (2013) [33]	—
**Innovation**				
	**Patient**			
		Implementation of intervention is perceived as risk-free by user	Harrington et al (2014) [71], Dey et al (2016) [74], Hayashi et al (2017) [76]	—
		Provision of warning/alert/reminder based on parameters monitored	Reese et al (2017) [35], McGillicuddy et al (2013) [36], van Lint et al (2015) [73]	—
		Provision of real-time feedback (eg, amount of dietary intake, blood pressure value) based on patients’ input	Sevick et al (2005) [60], Stark et al (2011) [63], Connelly et al (2012) [64], Hayashi et al (2017) [76]	—
		Perceived quality of eHealth intervention is excellent	—	Rifkin et al (2013) [33], McGillicuddy et al (2013) [36], Berman et al (2011) [59], Harrington et al (2014) [71]
	**Patient and care provider**			
		Interventions are compatible with existing work procedures	Rifkin et al (2013) [33]	—
		Implementation of intervention is perceived as advantageous by patient and care providers considering increasing access to health care services	Whitten et al (2008) [62]	—
		High acceptability of eHealth	Rifkin et al (2013) [33]	—
		Perceived quality of eHealth intervention is excellent	Gallar et al (2007) [61]	—
**Innovation strategies (patient and care provider)**				
	Well planned/structured implementation process		Liu et al (2017) [77]	—

^a^eHealth: electronic health.

^b^Not applicable.

#### Description of Effect Outcome Indicators

The effect outcome indicators most frequently reported were laboratory tests (eg, serum albumin, C-reactive protein; 6/23 articles) and blood pressure (BP; 5/23 articles). Interdialytic weight gain (4/23 articles), QoL (4/23 articles), and medication adherence (4/23 articles) were also frequently reported. Finally, 2 studies assessed effects on morbidity and mortality, 2 evaluated changes in medical cost, and 1 performed a cost-effectiveness analysis.

Out of 5 studies, 4 [36,68,70,75] reported a statistically significant positive effect on BP. Of the 2 studies [59,61] that evaluated changes in medical costs, 1 [59] reported a significant reduction in costs in the intervention group. A study reported an incremental cost-effectiveness ratio of US $175, showing that the implementation of a website-based self-management intervention for CKD patients was superior, considering effects and costs, to usual care [70]. Out of 3 studies, 2 [59,61] reported statistically significant improvements in hospitalization rates and emergency room visits. Out of 4 studies, 3 [35,36,66] reported statistically significant improvements in patients’ medication adherence. Out of 4 studies, 1 [70] reported a statistically significant improvement on QoL.

#### Description of Process Outcome Indicators

The process outcome indicator *satisfaction* was reported in one-third of included studies. A total of 2 studies [58,75] used interviews to evaluate satisfaction in patients or care providers. Patients were reported to be satisfied with the use of at-home telehealth and appreciated its utility in managing their health [58]. Patients using a smartphone-based self-management system indicated feeling more confident and more in control of their condition; the nurses found that the system helped prioritize patients who needed more attention [75]. A total of 5 studies used questionnaires to evaluate satisfaction of patients [36,71,72,74,76]. These studies reported patients were highly satisfied with eHealth self-management interventions.

Acceptability was also frequently reported and mostly measured using questionnaires, retention rates, or system data [33,36,69,74-76] (6/23 articles). All these studies reported that eHealth self-management interventions were acceptable to patients [33,36,69,74-76] and care providers [33,36]. Other process outcome indicators (including adherence to the intervention, first entry, length of dwell time, self-efficacy, perceived benefits, perceived control, and recorded errors) were less frequently used.

#### Description of Implementation Determinants

All but 4 studies [34,65,66,68] reported on determinants of implementation. Studies included used various methods (eg, qualitative interview and quantitative data analysis) to evaluate determinants of implementation. The determinant *ability of health care professionals to monitor and, if necessary, anticipate on patient measurements online* is mostly reported to make patients feel safe while using eHealth interventions [77], thereby influencing patients’ medication adherence [35] and adherence to interventions [35,73]. Moreover, *availability of sufficient skills/knowledge* [58,69,72] was reported as an important determinant to patients’ use of the eHealth self-management interventions. In addition, the determinant *provision of real-time feedback based on patients’ input* was frequently reported to influence patients’ adherence to self-monitoring and healthy behaviors [60,63,64,76]. The determinant *perceived quality of eHealth intervention is excellent* [61] was cited to influence both patients’ and care providers’ use of the intervention. The percent agreement between the 2 reviewers’ classification of the implementation determinants reported following the Fleuren framework was 76%, which is considered acceptable [48]. Discrepancies in classification were discussed until consensus was reached.

## Discussion

### Principal Findings

The main findings and implications have been presented in Textbox 3.

The evidence regarding the implementation and effectiveness of eHealth self-management interventions for CKD patients was reviewed. The 23 studies included were appraised on methodological quality, and all relevant data were extracted. Although the evidence base is still inconclusive, our review provides an indication that eHealth self-management interventions have the potential to improve CKD patients’ management and health outcomes. Furthermore, high acceptability of and satisfaction with the eHealth interventions used were reported. Owing to the heterogeneity of the intervention components and outcomes measures used, we could not determine which intervention components contributed most to the effects found. The determinant *ability of health care professionals to monitor and, if necessary, anticipate on patient measurements online* was most frequently reported to influence implementation. The determinants reported were not quantified, and the relative importance of each determinant could not be determined.

**Main findings and implications for this study.**

- Although the evidence base is still inconclusive, a majority of studies on electronic health (eHealth) self-management interventions report improvements on proximal outcomes (eg, blood pressure controlling) and mixed effects for more distal (eg, quality of life) outcomes.
- Evidence on the process level is more established; eHealth self-management interventions for chronic kidney disease patients are reported to be highly feasible, usable, and acceptable.
- To adequately assess eHealth intervention effect, future researchers should carefully consider their choice of outcomes (distal vs proximal) based on their sensitivity to capture meaningful change.
- Standardization of research design and methods in the evaluation of eHealth self-management interventions for chronic kidney disease patients is needed to optimize quality and comparability across studies and further elucidate which intervention components alone or in interaction contribute to the promising results found.

### Comparison of Findings

Most studies reported the evaluation of effect outcome indicators. The positive effects on patients’ BP controlling [36,68,70,75] and medication adherence [35,36,66] were consistently reported; no adverse outcomes were reported. These findings correspond with another review on eHealth interventions in CKD [39]. Compared with standard outpatient-based management, eHealth self-management interventions have the potential to reduce health care delivery costs [78]. Although this potential reduction in costs is essential for policy makers and clinicians to adopt eHealth self-management interventions, health care expenditures were only assessed in 3 of the studies included, with only 1 performing a cost-effectiveness analysis [70]. Hence, we cannot yet determine if and how these interventions might reduce medical costs. This finding is consistent with similar reviews, which conclude that studies on the cost-effectiveness of eHealth self-management interventions are either conflicting or lacking [32,54]. As evidence on cost-effectiveness is important to support the potential scale-up of eHealth technology, further research is needed to broaden this evidence base. Regarding QoL, only 1 out of 4 studies reported a significant improvement. A possible explanation for this finding was the short follow-up period instated to capture changes in a distal outcome such as QoL [59]. As QoL in CKD is an independent predictor of mortality and hospitalization [79,80], and thus important to evaluate, we advise further research to assess QoL with a longer follow-up period.

In general, we found that eHealth self-management interventions were reported to be highly feasible, usable, and acceptable. However, we found great diversity in the use and operationalization of outcome indicators and how they were measured. For instance, a study reported acceptability by measuring adoption, adherence to the recommended intervention use, user satisfaction, and feature usage [75]. In contrast, other studies [33,36] measured acceptability by asking patients “how acceptable they found the intervention” using a self-report scale. It is also notable that only 4 studies assessed implementation adherence, although finding no or limited intervention effects can be strongly related to patients’ nonadherence to eHealth interventions as prescribed [81,82]. Examining implementation adherence can help resolve the *black box* of patients’ adoption and continued use of the intervention, thereby preventing a type 3 error [83]. To tackle these issues, we advise researchers to use a standardized operationalization of process outcome indicators and measure implementation adherence to enable reliable interpretation of the intervention effect found.

Considering which outcomes are most sensitive to change is important. As eHealth interventions studies are mostly of short duration, they may not detect changes in distal outcomes (eg, QoL). Hence, effectivity might be easier to detect when proximal outcomes, close to the intervention strategies, are measured. For example, BP controlling can be an outcome sensitive to change if self-monitoring is the main intervention component. Functional outcomes (such as days needed to return to work), which can quantify patients’ subjective perceptions of the effect of treatment on their daily life, might also be very sensitive to change by eHealth interventions [84,85]. Moreover, researchers should consider if their outcomes reflect meaningful change and provide a clear rationale for their choice of laboratory parameters. For example, using serum albumin as an indicator for dietary adherence might be of limited value as it is influenced by other CKD characteristics (eg, low dialysis dose) [60]. 

Furthermore, improving knowledge on the effect modifiers at play in eHealth self-management interventions for CKD patients is important. None of the included studies provided detail on potentially relevant effect modifiers. We can identify some possible modifying factors based on research focusing on self-management interventions in other chronic, noncommunicable diseases (NCDs). For instance, a longer intervention duration might positively modify the effect of self-management interventions [86]. In addition, the patients’ health literacy level might modify intervention effect [87]. Self-management interventions for NCDs are mostly based on similar intervention principles and behavior change techniques. Moreover, the characteristics of patients suffering from NCDs are often similar. We, therefore, argue that the modifiers found to influence the outcomes of self-management interventions for NCDs in general might also be applicable for similar interventions targeting CKD patients. However, more research is needed to identify effect modifiers to self-management interventions targeting CKD and explore possible strategies to impact these factors.

### Electronic Health Self-Management Interventions

A large variety of eHealth self-management intervention components were used in the included studies (eg, self-monitoring and education), and the results differed greatly. These findings make it difficult and possibly premature to formulate a potentially ideal palette of eHealth self-management intervention components for CKD patients. However, reviewing results make it possible to identify which intervention components might be more promising than others. For instance, self-monitoring and the use of messages or alerts to nudge patient toward displaying healthy behaviors (see Multimedia Appendix 6) were most commonly reported as the effective components to optimize patient self-management skills.

Furthermore, few of the interventions studied were theory-based. The authors recommend that a strong theoretical foundation is necessary for the planning, design, evaluation, and implementation of eHealth self-management interventions [88]. We recommend building eHealth self-management interventions based on established behavior change techniques, such as formulated in the Behavior Change Techniques taxonomy [55]. Moreover, the use of cocreation methods and appreciative inquiry (such as described in the Center for eHealth Research and Disease Management [89] roadmap for eHealth development) can improve intervention fit with the needs and priorities expressed by professionals and patients.

### Determinants of Implementation

*Ability of health care professionals to monitor and, if necessary, anticipate patient measurements online* was reported as an important determinant of implementation. We argue that this ability of professionals to anticipate and act upon patient measurements might reduce patients’ feeling of isolation and/or anxiety caused by independently conducted treatments at home [77] and thereby increase patients’ adherence to implementation. In addition, *availability of sufficient skills/knowledge* was important for users to continue their use of eHealth technology. If participants are unfamiliar with the use of eHealth, this has been reported to limit their acceptance of eHealth interventions [58,69]. Proper training and tailored tutorials are needed to guide eHealth implementation to optimize knowledge and skills and promote intervention uptake [67,72]. The included studies used various methods to evaluate determinants of implementation. We suggest that future research should use validated tools for measuring implementation quality and related determinants, such as the Measurement Instrument for Determinants of Innovations questionnaire and Determinants of Implementation Behavior Questionnaire [90,91].

### Study Quality and Characteristics

Most studies were appraised to be of low to moderate quality. There is a heterogeneity of outcome measurement tools and reporting styles used in the articles included in this review. Therefore, we advise researchers to develop a more standardized approach to the use of outcome measures, guided by, for instance, the formulation of an International Consortium for Health Outcomes Measurement standard set for CKD [92]. In addition, we argue that detailed description and a thorough analysis of study design, methods, and intervention components used, based on a published theoretical framework such as Consolidated Standards of Reporting Trials-eHealth [93], can improve reporting and provide a basis for evaluating the validity and applicability of eHealth trials.

Data on eHealth self-management interventions for CKD patients in developing countries are still lacking, which corresponds with other reviews on eHealth interventions [94,95]. The need to perform such research in developing countries is high. eHealth interventions in these countries have the potential to improve the accessibility and cost-effectiveness of local care and ensure timely delivery of care to rural areas and diverse populations [20,24,96]. Furthermore, 9 studies had an intervention duration of fewer than 6 months. Few studies conducted a follow-up measurement. Forni Ogna et al [66] reported that the positive intervention effects were maintained only during the monitoring period; these effects had vanished 3 months after interruption of the drug adherence monitoring. This finding underlines that the effectiveness of eHealth self-management interventions should be tested during a longer study period and with follow-up measurements.

Of note, 3 studies with fewer than 10 participants were included. One might argue that such studies do not provide robust, generalizable evidence and should be excluded based only on their sample size. However, high-level evidence on the effectiveness of eHealth self-management interventions for CKD patients, for instance, generated by large RCTs, is very limited. Hence, studies with less robust designs are included, as in this stage, we feel that all evidence should be accumulated and taken into account as to broaden our view and deepen our understanding of the usability, implementability, and effectiveness of eHealth self-management interventions for CKD patients. Moreover, this decision is supported by similar systematic reviews on the effectivity of eHealth interventions that also included studies with smaller sample sizes [95,97,98]. That being said, results of this review should be interpreted with some caution.

### Strengths and Limitations

To our knowledge, this is the first systematic review to evaluate the entire spectrum of studies focusing on eHealth self-management interventions for CKD patients. Our review has some strengths. First, PRISMA guidelines were followed, and a robust search strategy was used in 8 databases. Second, a comprehensive analysis was conducted on the intervention components, outcome indicators, and determinants from the various studies. The kappa value and percent agreement obtained, and thus inter-rater reliability, showed that the validity of the appraisal could be considered fair. Finally, any discrepancies were discussed until consensus was reached.

Nevertheless, several limitations need to be addressed. First, as articles only published in English were included, some relevant articles might have been missed. Second, substantial heterogeneity of interventions and outcome measures made it difficult to draw firm conclusions about the evidence emerging from these studies, and results should be interpreted with caution.

### Conclusions

This review provides a comprehensive overview of studies evaluating eHealth self-management interventions for CKD patients. eHealth self-management interventions show promise to improve health outcomes in CKD patients. To adequately assess eHealth intervention effect, future researchers should carefully consider their choice of outcomes (distal vs proximal) based on their sensitivity to capture meaningful change. Also, to enable the standard design and scale-up of effective eHealth self-management interventions for CKD patients, a more detailed understanding of which individual intervention components lead to health outcome improvement and which determinants of the implementation can promote adherence and satisfaction with care is needed.

## Appendix

Multimedia Appendix 1 Search strategy.

Multimedia Appendix 2 Major types of electronic health.

Multimedia Appendix 3 Electronic health self-management intervention components.

Multimedia Appendix 4 Study characteristics.

Multimedia Appendix 5 Effects and references of outcome indicators.

Multimedia Appendix 6 Most frequently recommended electronic health self-management intervention components.

## Abbreviations

BP: blood pressure
CCAT: Crowe Critical Appraisal Tool
CKD: chronic kidney disease
CRD: Centre for Reviews and Dissemination
eHealth: electronic health
GFR: glomerular filtration rate
NCDs: noncommunicable diseases
PRISMA: Preferred Reporting Items for Systematic Reviews and Meta-Analyses
QoL: quality of life
RCT: randomized controlled trial
