# Supplementary material for: Electronic Health Self-Management Interventions for Patients With Chronic Kidney Disease: Systematic Review of Quantitative and Qualitative Evidence
Source: J Med Internet Res. 2019 Nov 5;21(11):e12384. doi: 10.2196/12384 (PMC6864489; doi:10.2196/12384)
Supplement: Multimedia Appendix 1 [file jmir_v21i11e12384_app1.pdf]

Multimedia Appendix 1 Search strategy

Search strategy	Search terms
**A**	(("ehealth"[tw] OR "e-health"[tw] OR "mhealth"[tw] OR "m-health"[tw] OR "telehealth"[tw] OR electronic communication*[tw] OR "Internet"[mesh] OR "internet"[tw] OR "Telemedicine"[mesh] OR telemed*[tw] OR "digital"[tw] OR "online"[tw] OR digital*[tw] OR mobile*[tw] OR "webbased"[tw] OR "web-based"[tw] OR "remote communication"[tw] OR "remote computer"[tw] OR "remote computers"[tw] OR "remote consultation"[tw] OR "remote health care"[tw] OR "remote healthcare"[tw] OR "remote monitoring"[tw] OR "remote system"[tw] OR "remote systems"[tw] OR "remote technologies"[tw] OR "remote technology"[tw] OR "teleconsultation"[tw] OR teleconsult*[tw] OR "Reminder Systems"[mesh] OR "Reminder Systems"[tw] OR "Reminder System"[tw] OR "Reminder Device"[tw] OR "Reminder Devices"[tw] OR "reminder messages"[tw] OR "reminder message"[tw] OR "Telephone"[mesh] OR telephon*[tw] OR "phone"[tw] OR "phones"[tw] OR "Cell Phones"[tw] OR "Smartphone"[tw] OR "Text Messaging"[tw] OR "Cell Phone"[tw] OR "Smartphones"[tw] OR iphon*[tw] OR "Text Messaging"[tw] OR text messag*[tw] OR "texting"[tw] OR "Electronic Mail"[mesh] OR "Electronic Mail"[tw] OR e-mail*[tw] OR email*[tw] OR "Telecommunications"[mesh] OR "app"[tw] OR "apps"[tw] OR webapp*[tw] OR "SMS"[tw] OR "mass communication"[tw] OR "blogging"[tw] OR "blog"[tw] OR "weblog"[tw] OR "social media"[tw] OR twitter*[tw] OR facebook*[tw] OR webcast*[tw] OR "Webcasts as Topic"[Mesh] OR "information technology"[tw] OR "Computer Systems"[mesh] OR "Software"[mesh] OR "Informatics"[mesh] OR "Electronic Health Records"[mesh] OR "Information Systems"[mesh] OR "decision support system"[tw] OR "computer"[tw] OR "computers"[tw] OR computer*[tw] OR "electronic"[tw] OR "software"[tw] OR "informatics"[tw] OR "health record system"[tw] OR "medical record system"[tw] OR "patient record system"[tw] OR "information system"[tw] OR "monitoring system"[tw] OR "screening system"[tw] OR "surveillance system"[tw] OR "information technology"[tw] OR "internet"[tw] OR "web"[tw] OR "mobile"[tw] OR "cellular phone"[tw] OR "smart phone"[tw] OR "telemedicine"[tw] OR "tele-care"[tw] OR "telecare"[tw] OR "tele-monitoring"[tw] OR "telemonitoring"[tw] OR "website"[tw] OR "websites"[tw] OR "wireless"[tw] OR "personal digital assistant"[tw] OR "computer-assisted instruction"[tw] OR "multimedia"[tw] OR "remote consultation"[tw] OR "technology"[tw] OR "communication network"[tw] OR "communication networks"[tw] OR "social network"[tw] OR social network*[tw] OR "ipad"[tw] OR ipad*[tw] OR "telenursing"[tw] OR telenurs*[tw] OR "virtual community"[tw] OR "webpage"[tw] OR "webpages"[tw] OR "web application"[tw] OR "web applications"[tw] OR "web access"[tw] OR "teleconference"[tw] OR teleconferen*[tw] OR telecommunication*[tw] OR "short message service"[tw]) AND ("Renal Insufficiency, Chronic"[Mesh] OR "chronic kidney disease"[tw] OR "chronic kidney diseases"[tw] OR "chronic renal disease"[tw] OR "chronic renal diseases"[tw] OR "chronic kidney failure"[tw] OR "chronic renal failure"[tw] OR "frasier syndrome"[tw] OR "chronic kidney"[tw] OR "chronic renal"[tw] OR "CKD"[tw] OR (("Kidney Diseases"[mesh] OR "kidney disease"[tw] OR "kidney diseases"[tw] OR "renal disease"[tw] OR "renal diseases"[tw]) AND ("Chronic Diseases"[tw] OR "chronic"[tw] OR chronic*[tw])) OR "Kidney Diseases"[mesh] OR "kidney disease"[tw] OR "kidney injury"[tw] OR "kidney disorder"[tw] OR "kidney insufficiency"[tw] OR "kidney failure"[tw] OR "kidney transplantation"[tw] OR "kidney transplant"[tw] OR "renal disease"[tw] OR "renal injury"[tw] OR "renal disorder"[tw] OR "renal insufficiency"[tw] OR "renal failure"[tw] OR "renal transplantation"[tw] OR "renal transplant"[tw] OR "dialysis"[tw] OR "kidney diseases"[tw] OR "kidney injuries"[tw] OR "kidney disorders"[tw] OR "kidney transplants"[tw] OR "renal diseases"[tw] OR "renal injuries"[tw] OR "renal disorders"[tw] OR "renal transplants"[tw] OR "dialysis"[tw] OR "Renal Dialysis"[Mesh] OR "hemodialysis"[tw] OR "hemodialysis"[tw]) AND ("Self Care"[Mesh] OR "self management"[tw] OR "self care"[tw] OR "self administration"[tw] OR "self medication"[tw] OR self manag*[tw] OR "self monitoring"[tw] OR self monitor*[tw] OR self car*[tw] OR self administr*[tw] OR self medicat*[tw] OR "Self Efficacy"[Mesh] OR "self efficacy"[tw] OR self effic*[tw] OR "self guided"[tw] OR self guid*[tw] OR "self help"[tw] OR self help*[tw] OR self treat*[tw] OR "empowerment"[tw] OR empower*[tw] OR "Power (Psychology)"[Mesh] OR "Health Education"[Mesh] OR "health education"[tw] OR "patient education"[tw] OR "Patient Education as Topic"[Mesh] OR "Patient Participation"[Mesh] OR "Patient Participation"[tw] OR "Community Participation"[Mesh] OR "Community Participation"[tw] OR "consumer participation"[tw] OR "Activities of Daily Living"[Mesh] OR "Activities of Daily Living"[tw] OR "ADL"[tw]))
**B**	(("ehealth"[ti] OR "e-health"[ti] OR "mhealth"[ti] OR "m-health"[ti] OR "telehealth"[ti] OR electronic communication*[ti] OR "Internet"[majr] OR "internet"[ti] OR "Telemedicine"[majr] OR telemed*[ti] OR "digital"[ti] OR "online"[ti] OR digital*[ti] OR mobile*[ti] OR "webbased"[ti] OR "web-based"[ti] OR "remote communication"[tw] OR "remote computer"[tw] OR "remote computers"[tw] OR "remote consultation"[tw] OR "remote health care"[tw] OR "remote healthcare"[tw] OR "remote monitoring"[tw] OR "remote system"[tw] OR "remote systems"[tw] OR "remote technologies"[tw] OR "remote technology"[tw] OR "teleconsultation"[tw] OR teleconsult*[tw] OR "Reminder Systems"[majr] OR "Reminder Systems"[ti] OR "Reminder System"[ti] OR "Reminder Device"[ti] OR "Reminder Devices"[ti] OR "reminder messages"[ti] OR "reminder message"[ti] OR "Telephone"[majr] OR telephon*[ti] OR "phone"[ti] OR "phones"[ti] OR "Cell Phones"[ti] OR "Smartphone"[ti] OR "Text Messaging"[ti] OR "Cell Phone"[ti] OR "Smartphones"[ti] OR iphon*[ti] OR "Text Messaging"[ti] OR text messag*[ti] OR "texting"[ti] OR "Electronic Mail"[majr] OR "Electronic Mail"[ti] OR e-mail*[ti] OR email*[ti] OR "Telecommunications"[majr] OR "app"[ti] OR "apps"[ti] OR webapp*[ti] OR "SMS"[ti] OR "mass communication"[ti] OR "blogging"[ti] OR "blog"[ti] OR "weblog"[ti] OR "social media"[ti] OR twitter*[ti] OR facebook*[ti] OR webcast*[ti] OR "Webcasts as Topic"[majr] OR "information technology"[ti] OR "monitoring system"[ti] OR "screening system"[ti] OR "surveillance system"[ti] OR "information technology"[ti] OR "internet"[ti] OR "web"[ti] OR "mobile"[ti] OR "cellular phone"[ti] OR "smart phone"[ti] OR "telemedicine"[ti] OR "tele-care"[ti] OR "telecare"[ti] OR "tele-monitoring"[ti] OR "telemonitoring"[ti] OR "website"[ti] OR "websites"[ti] OR "wireless"[ti] OR "personal digital assistant"[ti] OR "computer-assisted instruction"[ti] OR "multimedia"[ti] OR "remote consultation"[ti] OR "social network"[ti] OR social network*[ti] OR "ipad"[ti] OR ipad*[ti] OR "telenursing"[ti] OR telenurs*[ti] OR "virtual community"[ti] OR "webpage"[ti] OR "webpages"[ti] OR "web application"[ti] OR "web applications"[ti] OR "web access"[ti] OR "teleconference"[ti] OR teleconferen*[ti] OR telecommunication*[ti] OR "short message service"[ti]) AND ("Renal Insufficiency, Chronic"[majr] OR "chronic kidney disease"[ti] OR "chronic kidney diseases"[ti] OR "chronic renal disease"[ti] OR "chronic renal diseases"[ti] OR "chronic kidney failure"[ti] OR "chronic renal failure"[ti] OR "frasier syndrome"[ti] OR "chronic kidney"[ti] OR "chronic renal"[ti] OR "CKD"[ti] OR (("Kidney Diseases"[majr] OR "kidney disease"[ti] OR "kidney diseases"[ti] OR "renal disease"[ti] OR "renal diseases"[ti]) AND ("Chronic Diseases"[ti] OR "chronic"[ti] OR chronic*[ti])) OR "Kidney Diseases"[majr] OR "kidney disease"[ti] OR "kidney injury"[ti] OR "kidney disorder"[ti] OR "kidney insufficiency"[ti] OR "kidney failure"[ti] OR "kidney transplantation"[ti] OR "kidney transplant"[ti] OR "renal disease"[ti] OR "renal injury"[ti] OR "renal disorder"[ti] OR "renal insufficiency"[ti] OR "renal failure"[ti] OR "renal transplantation"[ti] OR "renal transplant"[ti] OR "dialysis"[ti] OR "kidney diseases"[ti] OR "kidney injuries"[ti] OR "kidney disorders"[ti] OR "kidney transplants"[ti] OR "renal diseases"[ti] OR "renal injuries"[ti] OR "renal disorders"[ti] OR "renal transplants"[ti] OR "dialysis"[ti] OR "Renal Dialysis"[majr] OR "hemodialysis"[ti] OR "hemodialysis"[ti]) AND ("intervention"[ti] OR "education"[ti] OR "information"[ti] OR train[ti] OR coach[ti] OR skills[ti] OR program[ti] OR counsel[ti] OR support[ti] OR "health promotion"[majr] OR "health promotion"[ti] OR

	"life style"[majr] OR "life style"[ti] OR "lifestyle"[ti] OR advocacy[ti] OR communicate[ti] OR empower[ti] OR "therapy"[ti] OR "therapeutics"[majr] OR "therapeutics"[ti] OR intensive[ti] OR manage[ti] OR "psychology"[ti] OR "psychology"[majr] OR "treat"[ti] OR "rehabilitation"[ti] OR "rehabilitation"[majr] OR "disease management"[ti] OR "disease management"[majr]) AND ("Patients"[Mesh] OR "patient"[tw] OR "patients"[tw] OR "inpatient"[tw] OR "inpatients"[tw] OR "outpatient"[tw] OR "outpatients|"[tw]))

**Database**	**Search**	**Result: number of hits**
PubMed	search A or B	995
Embase	search A or B	1198
Web of Science	search A or B	421
COCHRANE Library	search A or B	147
Emcare	search A or B	396
PsycINFO:	search A	79
Academic Search Premier: search	search A	23
ScienceDirect	search A	48
