# Supplementary material for: Electronic Health Self-Management Interventions for Patients With Chronic Kidney Disease: Systematic Review of Quantitative and Qualitative Evidence
Source: J Med Internet Res. 2019 Nov 5;21(11):e12384. doi: 10.2196/12384 (PMC6864489; doi:10.2196/12384)
Supplement: Multimedia Appendix 2 [file jmir_v21i11e12384_app2.pdf]

Multimedia Appendix 2 Major types of electronic health

- **Personal digital assistant (PDA)**: software/application/short message service (SMS) used to transmit patient data to physician/researcher.
- **Telemedicine**: a planned contact that is generally prearranged between a healthcare professional and a patient for the purpose of clinical consultation, advice, and treatment planning.
- **Computer**: computerized systems in which data are entered by the patient through the internet or are given to the patient by system.
- **Wearable devices**: worn or placed on a body part to record specific physiological changes (e.g., blood pressure monitors).
- **Multiple Component**: containing more than one eHealth technology.
