# Supplementary material for: Electronic Health Self-Management Interventions for Patients With Chronic Kidney Disease: Systematic Review of Quantitative and Qualitative Evidence
Source: J Med Internet Res. 2019 Nov 5;21(11):e12384. doi: 10.2196/12384 (PMC6864489; doi:10.2196/12384)
Supplement: Multimedia Appendix 3 [file jmir_v21i11e12384_app3.pdf]

Multimedia Appendix 3 Electronic health self-management intervention components

- **Education or training**: providing patients with training or educational materials on the use of eHealth (e.g., manual of device).
- **Plan/goals**: developing an action plan or setting personal goals (e.g., monitoring rates).
- **Self-monitoring**: using sensors or tools to track and record personal data (e.g., diet, blood pressure).
- **Interactive feedback from device**: feedback received from the device on recorded behavior or other personal data (e.g., nutrition analysis for daily diet from eHealth).
- **Message/alert to health caregivers**: the device provides health caregivers with (additional) information or an alert based on input/data received (e.g., warning of high blood pressure).
- **Message/alerts to patients from device**: the device provides patients with (additional) information or an alert based on input/data received (e.g., warning of high blood pressure).
- **Message/alert to patients from health caregivers**: health caregivers provide patients with (additional) information or an alert on the patients' device based on input/data received (e.g., warning of high blood pressure).
- **Quizzes**: providing quizzes about eHealth or health information to patients.
- **Counselling**: online communication between patients and care providers to improve self-management behaviors.
- **Daily use**: participants use eHealth every day.
