# Supplementary material for: Electronic Health Self-Management Interventions for Patients With Chronic Kidney Disease: Systematic Review of Quantitative and Qualitative Evidence
Source: J Med Internet Res. 2019 Nov 5;21(11):e12384. doi: 10.2196/12384 (PMC6864489; doi:10.2196/12384)
Supplement: Multimedia Appendix 4 [file jmir_v21i11e12384_app4.pdf]

Multimedia Appendix 4 Study characteristics

Study	Country	Study design	Focus	Participants	Sample size (baseline)	Age (years)	Recruitment strategy	Duration[a] (months)	Follow-up	Control condition
Minatodani et al (2013) [58]	United States	Mix-methods	Acceptability	HD	33	37-87	Hemodialy-sis center	NS	NS	NS
Berman et al (2011) [59]	United States	RCT (pilot study)	Health outcomes	HD	44	32-85	Hemodialy-sis center (outpatient)	21	NS	Usual care
Sevick et al (2005) [60]	United States	Single case	Effectiveness	HD[b]	6	63-70	Dialysis center (dietitian)	4	NS[c]	NS
Gallar et al (2007) [61]	Spain	NS	Use	PD[d] (CAPD[e] or automated peritoneal dialysis)	57	Mean: 44	Hospital (current cases)	3-24	NS	Without telemedicine
Whitten et al (2008) [62]	United States	NS	Clinical effects and perception	HD patients and providers	34 patients and 4 providers	NS	Dialysis center	22	NS	NS
Stark et al (2011) [63]	United States	RCT[f]	Self-monitoring rates	PD and HD	22 HD and 26 PD	Mean: PD: 56 and HD:51.7	Dialysis clinic (dietitian)	4	Per week	NS
Connelly et al (2012) [64]	United States	RCT (pilot study)	Design and evaluation	HD	18	Mean: 53	Face-to-face from 2 dialysis facilities (in-person)	1.5	NS	NS
Diamantidis et al (2013) [65]	United States	Prospect-ive cohort	Acceptance and usability	CKD[g]	108	Mean: 64	Medical system and clinics	12	6 months	NS

Forni Ogna et al (2013) [66]	Switzerland	RCT	Medicine adherence	Maintenance hemodialysis	50	mean: IG[h]:59 and UG[i]:61	Dialysis facilities	6	Once every 2 months	Usual care
Heiden et al (2013) [67]	Denmark	Qualitative	Usability	CKD	5	27-86	NS	NS	NS	NS
McGillicuddy et al (2013) [36]	United States	RCT	Feasibility and acceptability	Kidney transplant	21	Mean: IG:42.4 and UG:57.6	Kidney transplant clinic	3	NS	Standard care
Neumann et al (2013) [68]	Germany	Prospective study	Interdialytic weight gain	HD	120	Mean: IG:65.7 and UG:66.5	dialysis center	3	NS	Without telemetric monitoring
Rifkin et al (2013) [33]	United States	RCT	Systolic blood pressure	Stage 3 or greater CKD	47	Mean: IG: 68.5 and UG:67.9	Clinic (enrollment interview)	6	≥6 months	Using home blood pressure cuff
Welch et al (2013) [69]	United States	Comparative (pilot study)	Feasibility	HD	44	23-80	Outpatient dialysis unit	1.5	2 months	Using daily activity monitor application
Blakeman et al (2014) [70]	United Kingdom	RCT	Health outcomes	Stage 3 CKD	436	Mean: 72.1	General practices	6	6 months	Usual care
Harrington et al (2014) [71]	United States	Noncomparative (pilot study)	Use	CAPD	6	24-70	Dialysis program	8	26-240 days	NS
Diamantidis et al (2015) [72]	United States	Comparative (feasibility study)	Feasibility	Predialysis CKD	20	47-76	Ongoing observational cohort	1	NS	Short message service

van Lint et al (2015) [73]	Netherl-ands	Nonco-mparative (pilot study)	Patient experiences	Kidney transplant	30	25-70	Hospital	3	After 12 weeks	NS
Dey et al (2016) [74]	United Kingdom	Nonco-mparative (pilot study)	Acceptability	PD	22	26-93	Clinic following focus group meetings or clinic visits	Over 15	NS	NS
Ishani et al (2016) [34]	United States	RCT	Health outcomes	CKD	601	Mean: 75.1	Clinic (mail or telephone)	12	Every 3 months	Attending CKD education class and follow-up
Ong et al (2016) [75]	Canada	Nonco-mparative (pilot study)	Acceptability	Stage 4 or 5 CKD	47	Mean: 59	Clinic (general information newsletter)	6	NS	NS
Hayashi et al (2017) [76]	Japan	Compar-ative (pilot study)	Feasibility and usability	HD for at least 2 years	20	Mean: 54.9	Outpatient HD facility	0.5	2 weeks	Non self-management recording system
Liu et al (2017) [77]	Australia	Mixed methods	Usability	HD patients and nurses	74 patients and 3 nurses	21-78	NS	Over 6	NS	NS
Reese et al (2017) [35]	United States	RCT	Medicine adherence	Kidney transplant recipients	120	Mean: 50	Hospital	6	NS	Customized reminders and no reminders

[a]Duration: intervention duration.
[b]HD: hemodialysis.
[c]NS: not specified.
[d]PD: peritoneal dialysis.
[e]CAPD: continuous ambulatory peritoneal dialysis.
[f]RCT: randomized controlled trial.

[g]CKD: chronic kidney disease.
[h]IG: intervention group.
[i]UG: usual care group.

References

33. Rifkin DE, Abdelmalek JA, Miracle CM, Low C, Barsotti R, Rios P, et al. Linking clinic and home: a randomized, controlled clinical effectiveness trial of real-time, wireless blood pressure monitoring for older patients with kidney disease and hypertension. Blood Press Monit 2013 Feb;18(1):8-15 [FREE Full text] [doi: 10.1097/MBP.0b013e32835d126c] [Medline: 23275313]

34. Ishani A, Christopher J, Palmer D, Otterness S, Clothier B, Nugent S, Center for Innovative Kidney Care. Telehealth by an interprofessional team in patients with CKD: a randomized controlled trial. Am J Kidney Dis 2016 Jul;68(1):41-49. [doi: 10.1053/j.ajkd.2016.01.018] [Medline: 26947216]

35. Reese PP, Bloom RD, Trofe-Clark J, Mussell A, Leidy D, Levsky S, et al. Automated reminders and physician notification to promote immunosuppression adherence among kidney transplant recipients: a randomized trial. Am J Kidney Dis 2017 Mar;69(3):400-409. [doi: 10.1053/j.ajkd.2016.10.017] [Medline: 27940063]

36. McGillicuddy JW, Gregoski MJ, Weiland AK, Rock RA, Brunner-Jackson BM, Patel SK, et al. Mobile health medication adherence and blood pressure control in renal transplant recipients: a proof-of-concept randomized controlled trial. JMIR Res Protoc 2013 Sep 4;2(2):e32 [FREE Full text] [doi: 10.2196/resprot.2633] [Medline: 24004517]

58. Minatodani DE, Chao PJ, Berman SJ. Home telehealth: facilitators, barriers, and impact of nurse support among high-risk dialysis patients. Telemed J E Health 2013 Aug;19(8):573-578. [doi: 10.1089/tmj.2012.0201] [Medline: 23742629]

59. Berman SJ, Wada C, Minatodani D, Halliday T, Miyamoto R, Lindo J, et al. Home-based preventative care in high-risk dialysis patients: a pilot study. Telemed J E Health 2011 May;17(4):283-287. [doi: 10.1089/tmj.2010.0169] [Medline: 21480788]

60. Sevick MA, Piraino B, Sereika S, Starrett T, Bender C, Bernardini J, et al. A preliminary study of PDA-based dietary self-monitoring in hemodialysis patients. J Ren Nutr 2005 Jul;15(3):304-311. [doi: 10.1016/j.jrn.2005.04.003] [Medline: 16007560]

61. Gallar P, Vigil A, Rodriguez I, Ortega O, Gutierrez M, Hurtado J, et al. Two-year experience with telemedicine in the follow-up of patients in home peritoneal dialysis. J Telemed Telecare 2007;13(6):288-292. [doi: 10.1258/135763307781644906] [Medline: 17785025]

62. Whitten P, Buis L. Use of telemedicine for haemodialysis: perceptions of patients and health-care providers, and clinical effects. J Telemed Telecare 2008;14(2):75-78. [doi: 10.1258/jtt.2007.070411] [Medline: 18348752]

63. Stark S, Snetselaar L, Piraino B, Stone RA, Kim S, Hall B, et al. Personal digital assistant-based self-monitoring adherence rates in 2 dialysis dietary intervention pilot studies: BalanceWise-HD and BalanceWise-PD. J Ren Nutr 2011 Nov;21(6):492-498 [FREE Full text] [doi: 10.1053/j.jrn.2010.10.026] [Medline: 21420316]

64. Connelly K, Siek KA, Chaudry B, Jones J, Astroth K, Welch JL. An offline mobile nutrition monitoring intervention for varying-literacy patients receiving hemodialysis: a pilot study examining usage and usability. J Am Med Inform Assoc 2012;19(5):705-712 [FREE Full text] [doi: 10.1136/amiajnl-2011-000732] [Medline: 22582206]

65. Diamantidis CJ, Fink W, Yang S, Zuckerman MR, Ginsberg J, Hu P, et al. Directed use of the internet for health information by patients with chronic kidney disease: prospective cohort study. J Med Internet Res 2013 Nov 15;15(11):e251 [FREE Full text] [doi: 10.2196/jmir.2848] [Medline: 24240617]

66. Ogna VF, Pruijm M, Zweiacker C, Wuerzner G, Tousset E, Burnier M. Clinical benefits of an adherence monitoring program in the management of secondary hyperparathyroidism with cinacalcet: results of a prospective randomized controlled study. Biomed Res Int 2013;2013:104892 [FREE Full text] [doi: 10.1155/2013/104892] [Medline: 23971019]

67. Heiden S, Buus AA, Jensen MH, Hejlesen OK. A diet management information and communication system to help chronic kidney patients cope with diet restrictions. Stud Health Technol Inform 2013;192:543-547. [doi: 10.3233/978-1-61499-289-9-543] [Medline: 23920614]

68. Neumann CL, Wagner F, Menne J, Brockes C, Schmidt-Weitmann S, Rieken EM, et al. Body weight telemetry is useful to reduce interdialytic weight gain in patients with end-stage renal failure on hemodialysis. Telemed J E Health 2013 Jun;19(6):480-486. [doi: 10.1089/tmj.2012.0188] [Medline: 23614336]

69. Welch JL, Astroth KS, Perkins SM, Johnson CS, Connelly K, Siek KA, et al. Using a mobile application to self-monitor diet and fluid intake among adults receiving hemodialysis. Res Nurs Health 2013 Jun;36(3):284-298 [FREE Full text] [doi: 10.1002/nur.21539] [Medline: 23512869]

70. Blakeman T, Blickem C, Kennedy A, Reeves D, Bower P, Gaffney H, et al. Effect of information and telephone-guided access to community support for people with chronic kidney disease: randomised controlled trial. PLoS One 2014;9(10):e109135 [FREE Full text] [doi: 10.1371/journal.pone.0109135] [Medline: 25330169]

71. Harrington DM, Myers L, Eisenman K, Bhise V, Nayak KS, Rosner MH. The use of a tablet computer platform to optimize the care of patients receiving peritoneal dialysis: a pilot study. Blood Purif 2014;37(4):311-315. [doi: 10.1159/000365440] [Medline: 25170838]

72. Diamantidis CJ, Ginsberg JS, Yoffe M, Lucas L, Prakash D, Aggarwal S, et al. Remote usability testing and satisfaction with a mobile health medication inquiry system in CKD. Clin J Am Soc Nephrol 2015 Aug 7;10(8):1364-1370 [FREE Full text] [doi: 10.2215/CJN.12591214] [Medline: 26220816]

73. van Lint CL, van der Boog PJ, Wang W, Brinkman WP, Rövekamp TJ, Neerincx MA, et al. Patient experiences with self-monitoring renal function after renal transplantation: results from a single-center prospective pilot study. Patient Prefer Adherence 2015;9:1721-1731 [FREE Full text] [doi: 10.2147/PPA.S92108] [Medline: 26673985]
74. Dey V, Jones A, Spalding EM. Telehealth: acceptability, clinical interventions and quality of life in peritoneal dialysis. Sage Open Med 2016;4:2050312116670188 [FREE Full text] [doi: 10.1177/2050312116670188] [Medline: 27757228]
75. Ong SW, Jassal SV, Miller JA, Porter EC, Cafazzo JA, Seto E, et al. Integrating a smartphone-based self-management system into usual care of advanced CKD. Clin J Am Soc Nephrol 2016 Jun 6;11(6):1054-1062 [FREE Full text] [doi: 10.2215/CJN.10681015] [Medline: 27173169]
76. Hayashi A, Yamaguchi S, Waki K, Fujiu K, Hanafusa N, Nishi T, et al. Testing the feasibility and usability of a novel smartphone-based self-management support system for dialysis patients: a pilot study. JMIR Res Protoc 2017 Apr 20;6(4):e63 [FREE Full text] [doi: 10.2196/resprot.7105] [Medline: 28428168]
77. Liu N, Kim J, Jung Y, Arisy A, Nicdao MA, Mikaheal M, et al. Remote monitoring systems for chronic patients on home hemodialysis: field test of a copresence-enhanced design. JMIR Hum Factors 2017 Aug 29;4(3):e21 [FREE Full text] [doi: 10.2196/humanfactors.7078] [Medline: 28851680]
