# Supplementary material for: Electronic Health Self-Management Interventions for Patients With Chronic Kidney Disease: Systematic Review of Quantitative and Qualitative Evidence
Source: J Med Internet Res. 2019 Nov 5;21(11):e12384. doi: 10.2196/12384 (PMC6864489; doi:10.2196/12384)
Supplement: Multimedia Appendix 5 [file jmir_v21i11e12384_app5.pdf]

Multimedia Appendix 5: Effects and references of outcome indicators

Blood pressure 4
[36] McGillicuddy *et al.* (2013) 4
[68] Neumann *et al*. (2013) 5
[70] Blakeman *et al*. (2014) 5
[75] Ong *et al*. (2016) 6
[33] Rifkin *et al*. (2013) 6
Quality of life 7
[70] Blakeman *et al*. (2014) 7
[59] Berman *et al*. (2011) / [58] Minatodani *et al*. (2013) 7
[74] Dey *et al*. (2016) 8
[76] Hayashi *et al*. (2017) 8
Laboratory tests 9
[66] Forni Ogna *et al*. (2013) 9
[68] Neumann et al. (2013) 9
[60] Sevick *et al*. (2005) 10
[62] Whitten *et al*. (2008) 10
[75] Ong *et al*. (2016) 11
[76] Hayashi *et al*. (2017) 11
IWG 12
[68] Neumann *et al*. (2013) 12
[60] Sevick *et al*. (2005) 12
[69] Welch *et al*. (2013) 13
[76] Hayashi *et al*. (2017) 13
Morbidity, mortality 14
[34] Ishani *et al*. (2016) 14
[61] Gallar *et al*. (2007) 14
Hospitalization rate, emergency room visits 15
[61] Gallar *et al*. (2007) 15
[59] Berman *et al*. (2011) / [66] Minatodani *et al*. (2013) 15
[34] Ishani *et al*. (2016) 16
Medical cost 17
[59] Berman *et al*. (2011) / [66] Minatodani *et al*. (2013) 17
[61] Gallar *et al*. (2007) 17

Cost effectiveness ... 18
[70] Blakeman *et al*. (2014) ... 18
Nutrition, dietary intake ... 19
[62] Whitten *et al*. (2008) ... 19
[69] Welch *et al*. (2013) ... 19
Medication adherence ... 20
[35] Reese *et al*. (2017) ... 20
[36] McGillicuddy *et al*. (2013) ... 20
[66] Forni Ogna *et al*. (2013) ... 20
[33] Rifkin *et al*. (2013) ... 21
Acceptability ... 22
[69] Welch *et al*.(2013) ... 22
[74] Dey *et al*. (2016) ... 22
[76] Hayashi *et al*. (2017) ... 22
[33] Rifkin *et al*. (2013) ... 23
[36] McGillicuddy *et al*. (2013) ... 23
[75] Ong *et al*. (2016) ... 23
Usability ... 24
[64] Connelly *et al*. (2012) ... 24
[67] Heiden *et al*. (2013) ... 24
[76] Hayashi *et al*. (2017) ... 24
[62] Whitten *et al*. (2008) ... 25
[77] Liu *et al*. (2017) ... 25
Satisfaction ... 26
[36] McGillicuddy *et al*. (2013) ... 26
[59] Berman *et al*. (2011) / [58] Minatodani *et al*. (2013) ... 26
[71] Harrington *et al*. (2014) ... 26
[72] Diamantidis *et al*. (2015) ... 26
[73] van Lint *et al*. (2015) ... 27
[74] Dey *et al*. (2016) ... 27
[76] Hayashi *et al*. (2017) ... 27
[75] Ong *et al*. (2016) ... 27
Adherence to intervention ... 28
[35] Reese *et al*. (2017) ... 28

[63] Stark *et al*. (2011) 28

[73] van Lint *et al*. (2015) 29

[75] Ong *et al*. (2016) 29

First entry, length of dwell time 30

[65] Diamantidis *et al*. (2013) 30

Self-efficacy 31

[69] Welch *et al*. (2013) 31

Perceived benefits 32

[69] Welch *et al*. (2013) 32

Perceived control 33

[69] Welch *et al*. (2013) 33

Recorded errors 34

[72] Diamantidis *et al*. (2015) 34

Blood pressure

[36] McGillicuddy *et al.* (2013)

Figure 3. SBP across time by treatment group (mean with Bonferroni 95% CI).

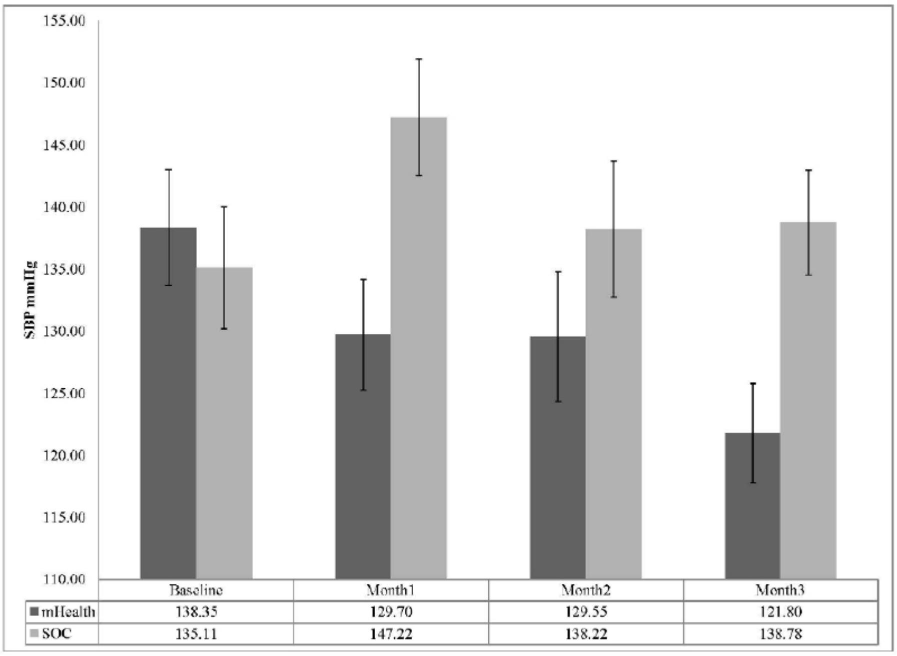

Figure 4. DBP across time by treatment group (mean with Bonferroni 95% CI).

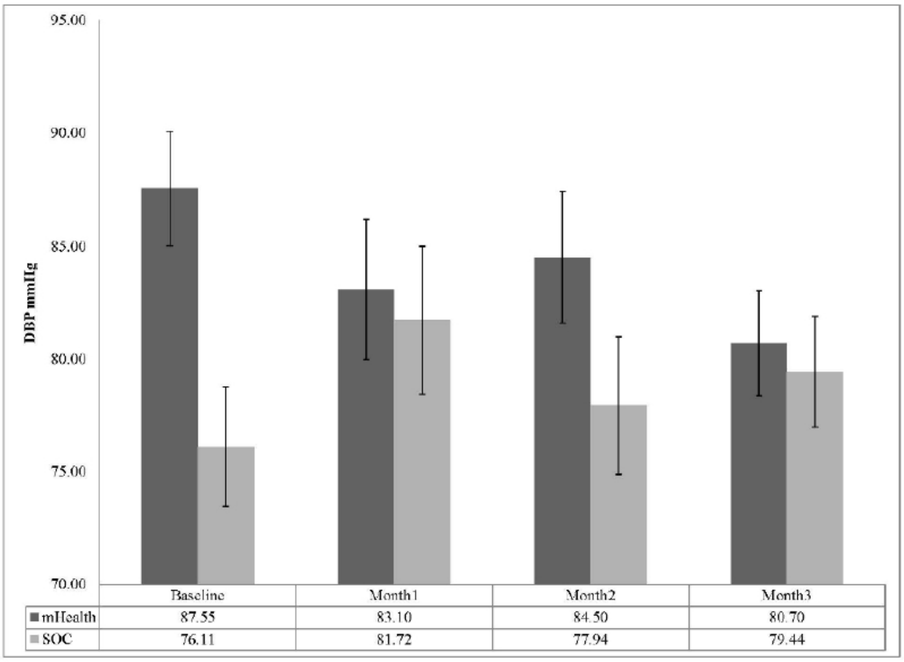

[68] Neumann *et al.* (2013)

Table 2. Blood Pressure

	Blood pressure (mm Hg)			
	Before beginning of study		End of study	
	TG	CG	TG	CG
At the beginning of dialysis				
IDI2				
Systolic	117.0 ± 18.9	116.9 ± 16.9	116.0 ± 17.0	117.9 ± 19.5
Diastolic	66.8 ± 10.2	67.0 ± 9.0	66.9 ± 8.7[a]	65.0 ± 8.8[a]
Unfiltered				
Systolic	118.3 ± 19.8	117.1 ± 17.4	117.7 ± 17.9	119.6 ± 18.9
Diastolic	67.5 ± 9.7	67.0 ± 9.5	67.8 ± 9.3[a]	66.4 ± 9.3[a]
At the end of dialysis				
IDI2				
Systolic	114.0 ± 17.1	113.1 ± 18.4	111.5 ± 14.3	110.7 ± 18.2
Diastolic	65.1 ± 8.4	64.1 ± 6.8	65.2 ± 7.1	64.6 ± 8.1
Unfiltered				
Systolic	113.4 ± 16.2[a]	111.7 ± 17.3[a]	112.7 ± 15.0	111.4 ± 17.2
Diastolic	65.2 ± 7.9	64.4 ± 7.3	65.6 ± 7.8[a]	64.0 ± 7.7[a]

Data are mean ± standard deviation values.

[a] $p < 0.05$.

CG, control group; IDI2, second interdialytic interval; TG, telemetry group.

[70] Blakeman *et al.* (2014)

Table 3. Results of regression analyses of primary and secondary outcomes at 6 months.

	Unadjusted mean (SD); N					
Outcome	BRIGHT intervention[1]	Usual care[1]	Adjusted mean difference[2] (95% CI)	P Value[2]	Effect size[2]	Effect size from complete cases analysis[1]
**Primary Outcomes**						
Blood pressure control	67.3%[3]; 193	55.3%[3]; 210	1.85[4] (1.25, 2.72)	0.002*	1.85[4] (1.25, 2.72)	1.90[4] (1.28, 2.83)
Positive and active engagement with life (heiQ) positive and active engagement in life *Higher score = higher engagement with life*	66.4 (19.7); 180	66.5 (17.6); 194	0.00 (−3.20, 3.21)	0.999	0.00 (−0.16, 0.16)	−0.01 (−0.17, 0.16)
Health-related Quality of Life (EQ-5D)	0.71 (0.28); 179	0.67 (0.29); 193	0.05 (0.01, 0.08)	0.027*	0.16 (0.03, 0.29)	0.18 (0.06, 0.30)

[75] Ong *et al.* (2016)

Table 2. Mean BP at the beginning and end of the study by site of assessment

Site, Patients, and Visit	No.	SBP Mean, mmHg	SD	DBP Mean, mmHg	SD
**Clinic**					
All					
Baseline	47	130.6	17.4	78.9	11.1
Exit	36	122.8	15.5	77.3	13.4
∂	36	−6.8		−0.9	
95% CI	36	−48.2 to 34.7		−19.4 to 17.7	
Uncontrolled hypertension					
Baseline	25	141.9	14.9	84.4	10.2
Exit	20	126.3	13.6	80.0	13.7
∂	20	−13.5		−2.6	
95% CI	20	−58.5 to 31.5		−22.1 to 17.0	
Normotensives					
Baseline	22	117.9	9.5	72.7	8.5
Exit	16	118.6	17.1	73.9	12.6
∂	16	1.6		1.2	
95% CI	16	−32.0 to 35.3		−17.3 to 19.8	
**Home**					
All					
Baseline	47	134.5	13.8	79.4	11.2
Exit	36	131.3	13.5	77.3	12.4
∂	36	−3.4		−2.1	
95% CI	36	−5.0 to −1.8		−2.9 to −1.2	
Uncontrolled hypertension					
Baseline	25	139.6	15.9	80.4	11.9
Exit	20	133.9	13.2	77.1	12.3
∂	20	−4.3		−1.5	
95% CI	20	−6.6 to −2.1		−2.6 to −0.4	
Normotensives					
Baseline	22	128.7	8.1	78.2	10.6
Exit	16	128.0	13.5	77.6	12.8
∂	16	−2.2		−2.8	
95% CI	16	−4.4 to −0.1		−4.2 to −1.5	

SBP, systolic BP; DBP, diastolic BP; ∂, change in BP readings from baseline to exit; 95% CI, 95% confidence interval.

[33] Rifkin *et al.* (2013)

Table 3 Comparison of telemonitoring and usual care groups at start and conclusion

	Telemonitoring	Usual care	*P* for two-group comparison
Systolic blood pressure (mmHg)			
Start of study	147±17.5	146±8.8	0.87
End of study	136±15.6	140±14.4	0.48
Change	−13 (0, −31); *P*=0.009 vs. start	−8.5 (0, −18); *P*=0.031 vs. start	0.32
Diastolic blood pressure (mmHg)			
Start of study	77±12.3	81±10.9	0.35
End of study	73±10.3	73±12.6	0.93
Change	−3.5 (−3, 12.5); *P*=0.02 vs. start	−8 (3, 13); *P*=0.0079 vs. start	0.35
Mean arterial pressure (mmHg)			
Start of study	101±11	102.9±9.6	0.46
End of study	93.9±8.6	95.2±11.7	0.67
Change	−7.13 (−16.5, 0.05)	−7.67 (−12.3, −1.7)	0.87
Creatinine (mg/dl)			
Start of study	2.13±0.66	1.96±0.53	0.42
End of study	2.17±0.76	2.32±0.84	0.64
Change	0.04 (−0.09, 0.34)	0.28 (0, 0.34)	0.12
eGFR (ml/min/1.73 m^2)			
Start of study	37.3±14.2	39.4±10.6	0.64
End of study	37.9±16.7	34.5±13.2	0.51
Change	0.6 (−3.4, 1.8)	−3.69 (−6.2, 0)	0.14
Total number of medications			
Start of study	11.2±4.1	11.1±5.6	0.91
End of study	12±4.6	12.8±5.1	0.62
Change	0.96 (0, 2)	1.53 (0, 3)	0.33
Number of blood pressure medications			
Start of study	3.9±1.4	3.9±1.4	0.92
End of study	4±1.2	3.9±1.3	0.61
Change	0.89 (−1, 4)	1 (0, 2)	0.91
Total number of interventions	4.6±2.1	1.7±0.97	<0.01
Morisky Medication Adherence Scale			
Start of study	6.9±1.3	6.4±1.7	0.35
End of study	7±1.2	7.2±1.4	0.58
Change	0.11 (0, 0.5)	0.67 (0, 2)	0.17

Data are presented as means±SD, median and IQR, or percentages.
Morisky Medication Adherence Scale, 8 represents perfect adherence, 6–7 represent medium adherence, 5 and below represent poor adherence.
eGFR, estimated glomerular filtration rate.

Quality of life

[70] Blakeman *et al*. (2014)

Table 3. Results of regression analyses of primary and secondary outcomes at 6 months.

Outcome	Unadjusted mean (SD); N<br>BRIGHT intervention[1]	Usual care[1]	Adjusted mean difference[2] (95% CI)	P Value[2]	Effect size[2]	Effect size from complete cases analysis[1]
**Primary Outcomes**						
Blood pressure control	67.3%[3]; 193	55.3%[3]; 210	1.85[4] (1.25, 2.72)	0.002*	1.85[4] (1.25, 2.72)	1.90[4] (1.28, 2.83)
Positive and active engagement with life (heiQ) positive and active engagement in life *Higher score = higher engagement with life*	66.4 (19.7); 180	66.5 (17.6); 194	0.00 (−3.20, 3.21)	0.999	0.00 (−0.16, 0.16)	−0.01 (−0.17, 0.16)
Health-related Quality of Life (EQ-5D)	0.71 (0.28); 179	0.67 (0.29); 193	0.05 (0.01, 0.08)	0.027*	0.16 (0.03, 0.29)	0.18 (0.06, 0.30)
**Secondary Outcomes**						

[59] Berman *et al*. (2011) / [58] Minatodani *et al*. (2013)

Table 1. Healthcare Resource Outcomes

	REMOTE TECHNOLOGY *N* = 19<br>MEAN (SD)	USUAL CARE *N* = 25<br>MEAN (SD)	*P*-VALUE
Gender			
Females	7	11	
Males	12	14	
Total study days per patient	353 (208.39)	334 (202.63)	0.381
Range	108–649	138–714	
Age	56.78 (12.01)	62.00 (14.46)	0.190
Range	42–79	32–85	
Risk score	1.41 (0.16)	1.35 (0.11)	0.199
Range	1.217–1.827	1.200–1.510	
Charlson comorbidity index (modified)[a]	3.35 (1.90)	3.00 (1.82)	0.560
Range	0–6	0–7	
Karnofsky score	58.75 (3.15)	57.60 (5.22)	0.433
Range	50–60	40–60	
SF-36 (preintervention)	63.90 (15.50)	59.13 (16.85)	0.170
Range	34.4–88.0	21.7–90.0	
SF-36 (6–9 months)	60.76 (20.70)	59.50 (17.56)	0.417
Range	21–98	18–89	
Hospital days (per study day)	0.0082 (0.0225)	0.0355 (0.0555)	0.016[b]
Range	0–0.0926	0–0.1842	
Hospitalizations (per study day)	0.0018 (0.0029)	0.0056 (0.0062)	0.008[b]
Range	0–0.0093	0–0.0205	
ER visits (per study day)	0.0003 (0.0008)	0.0019 (0.0036)	0.035[b]
Range	0–0.0030	0–0.0132	
Hospital ER charges (per study day)	$114 (179.45)	$322 (336.53)	0.041[b]
Range	$6.99–$597.56	$0–$1,096.96	

[a]Hemmelgarn et al.[6]
[b]$p < 0.05$.
ER, emergency room; SF-36, 36-Item Short Form Health Survey.

[74] Dey *et al.* (2016)

Table 3. QUEST and QOL scores at the start and end of programme.

	Initial	Final	p-Values
QUEST,[a] mean (SD)			
Device	4.5 (0.5)	4.5 (0.6)	
Service	4.2 (0.9)	4.1 (1.0)	
Total (device + service)	4.4 (0.5)	4.3 (0.6)	
QOL, mean (SD)[b]			
Symptom/problem list	70.2 (17.0)	64.7 (15.8)	0.27
Effects of kidney disease	75.6 (21.5)	69.6 (24.3)	0.37
Burden of kidney disease	55.1 (33.9)	40.9 (24.7)	0.49
SF-12 Physical Health Composite	29.7 (6.1)	31.5 (8.8)	0.54
SF-12 Mental Health Composite	46.2 (10.4)	43.6 (11.6)	0.43

QUEST: Quebec User Evaluation of Satisfaction with assistive Technology; SD: standard deviation; QOL: quality of life; SF-12: short form-12.
[a]Scores on a scale of 1–5 with 1 indicating *not satisfied at all* and 5 being *very satisfied*.
[b]Scores on a scale of 0–100 with higher scores indicating better quality of life.

[76] Hayashi *et al.* (2017)

Table 5. Comparison of changes in the Kidney Disease Quality of Life scores before and after the study period in the Self-Management and Recording System for Dialysis group (n=8).

Kidney Disease Quality of Life	Baseline score mean (SD)	Follow-up score mean (SD)	*P* value
Symptoms/problems	83.6 (7.8)	84.4 (8.4)	.84
Effect of kidney disease	78.4 (16.3)	79.7 (16.3)	.42
Burden of kidney disease	36.7 (21.8)	38.3 (16.5)	.79
Work status	75.0 (37.8)	75.0 (37.8)	>.99
Cognitive function	87.6 (11.8)	87.5 (12.6)	.52
Quality of social interaction	90.5 (10.1)	86.7 (19.2)	.20
Sleep	58.5 (7.5)	66.9 (18.1)	.16
Social support	68.7 (25.9)	62.5 (36.5)	.58
Dialysis staff encouragement	75.0 (11.6)	82.8 (14.8)	.10
Patient satisfaction	85.4 (16.5)	79.2 (21.3)	.20
Physical functioning	91.3 (7.4)	83.8 (20.0)	.28
Role functioning physical	78.1 (28.1)	84.4 (35.2)	.67
Bodily pain	65.3 (26.2)	74.1 (25.9)	.17
General health perception	52.9 (16.0)	54.4 (13.5)	.65
Vitality	56.3 (15.8)	65.0 (22.7)	.10
Social functioning	75.0 (20.0)	84.4 (14.6)	.048
Role functioning emotional	91.7 (15.4)	100 (0.0)	.17
Mental health	75.0 (16.1)	74.6 (16.)2	.96

Laboratory tests

[66] Forni Ogna *et al.* (2013)

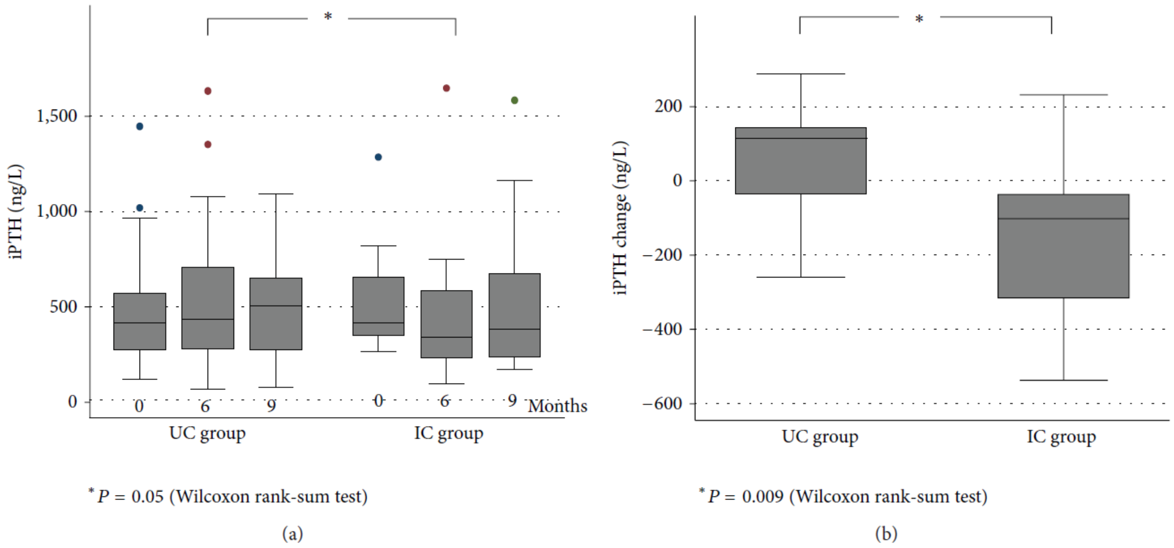

FIGURE 3: Absolute intact parathyroid hormone (iPTH) values (a) and iPTH mean changes (b). Note: conversion factors for units: iPTH in ng/L to pmol/L, divided by 9.43.

[68] Neumann et al. (2013)

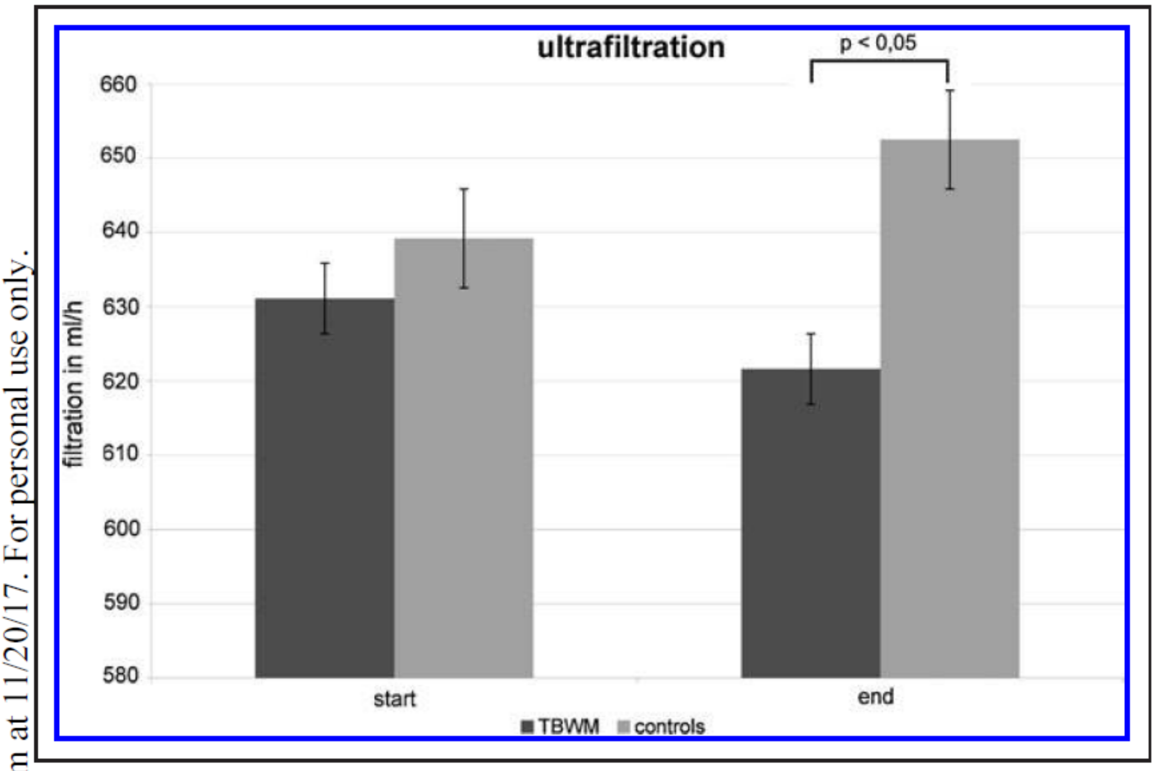

Fig. 4. Ultrafiltration at the end point (unfiltered). TBWM, telemetric body weight measurement.

rtpub.com at 11/20/17. For personal use only.

[60] Sevick *et al.* (2005)

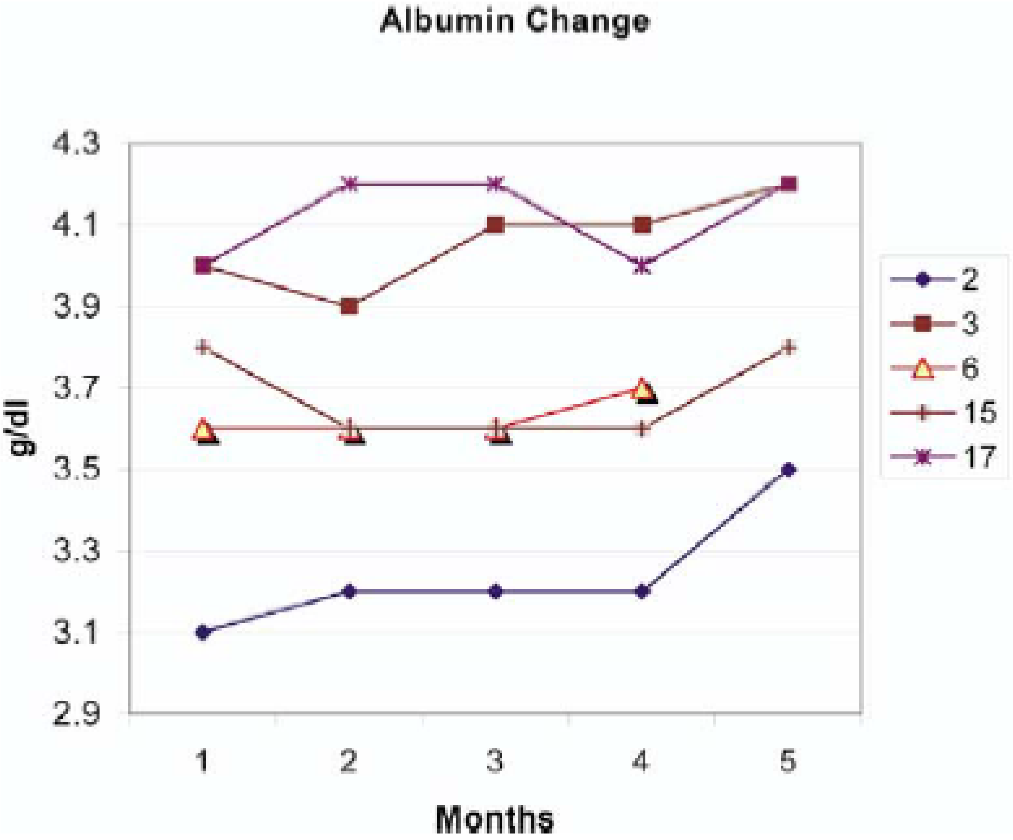

Figure 1. Monthly serum albumin levels.

[62] Whitten *et al.* (2008)

Table 3 Comparisons of clinical data to Renal Network 11 recommendations and best practices

	Renal Network 11 recommendation	Renal Network 11 best practices	Six-month average
Haemoglobin	≥80% of patients should have haemoglobin ≥11 g/dL	>85% of patients have haemoglobin ≥11 g/dL	89%[*,†]
Urea reduction ratio	≥80% of patients should have urea reduction ratio ≥65%	>85% of patients will have urea reduction ratio ≥65%	98%[*,†]
Albumin	≥80% of patients should have mean serum albumin ≥ lower limit of normal (3.4 g/dL)	>85% of patients have a mean serum albumin ≥ lower limit of normal (3.4 g/dL)	76%
	≤10% of patients should have mean serum album <0.9 lower limit of normal (3.1 g/dL)	≤10% of patients with a mean serum album <0.9 lower limit of normal (3.1 g/dL)	7%[*,†]
Phosphorus	≥70% of patients should have mean serum phosphorus ≤5.5 mg/dL	>80% of patients should have mean serum phosphorus ≤5.5 mg/dL	74%[*]
	<10% of patients should have mean serum phosphorus >8 mg/dL	<5% of patients should have mean serum phosphorus >8 mg/dL	0%[*,†]
Calcium	≥80% of patients should have mean corrected serum calcium concentration ≤10.2 mg/dL	>85% of patients should have mean corrected serum calcium concentration ≤10.2 mg/dL	91%[*,†]
		>75% of patients should have a mean corrected calcium concentration ≤9.5 mg/dL	33%

[*]Clinical data from investigation met Renal Network 11 recommendations
[†]Clinical data from investigation met Renal Network 11 recommendations for best practices

[75] Ong *et al*. (2016)

Table 4. Comparison of CKD-specific laboratory results between baseline and 6 months

Laboratory Values (Range)	Baseline, *n*=47	End, *n*=36	*P* Value
Serum potassium (3.2–5.0), mEq/L	4.7±0.5	4.6±0.6	0.18
Patients within range, %	78.7	80.6	0.22
Serum phosphate (<4.6), mg/dl	4.21±0.41	4.21±0.87	0.28
Patients within range, %	74.5	75	>0.99
Hemoglobin (10.0–12.0), g/dl	11.8±1.7	11.8±1.5	0.52
Patients within range, %	29.8	25	>0.99
Patients receiving ESA, %	40.4	52.8	0.51

ESA, erythropoietin-stimulating agent.

[76] Hayashi *et al*. (2017)

Multimedia Appendix 1 Table showing clinical parameters at baseline, study period and follow-up period in the Self-Management and Recording System for Dialysis group (n=9) and the non-Self-Management and Recording System for Dialysis group (n=11).

	Baseline	Study period	Follow-up period	
**SMART-D[a] group (n=9)**	mean (SD)			*P* value
IWG[b] (%DW[c]/day)	2.2 (0.4)	2.1 (0.3)	2.1 (0.4)	.68
Serum potassium concentrations (mEq/L)	4.9 (0.6)	5.1 (0.7)	5.0 (0.5)	.07
Serum phosphorus concentrations (mg/dL)	5.4 (1.2)	5.2 (1.0)	5.2 (1.2)	.67
**Non-SMART-D group (n=11)**	mean (SD)			*P* value
IWG (%DW/day)	2.2 (0.4)	2.3 (0.5)	2.4 (0.5)	.13
Serum potassium concentrations (mEq/L)	5.1 (0.8)	5.1 (0.6)	5.1 (0.7)	.84
Serum phosphorus concentrations (mg/dL)	5.0 (1.1)	5.0 (1.3)	5.2 (1.0)	.73

[a]SMART-D: Self-Management and Recording System for Dialysis.
[b]IWG: interdialysis weight gain.
[c]DW: dry weight.

IWG

[68] Neumann *et al.* (2013)

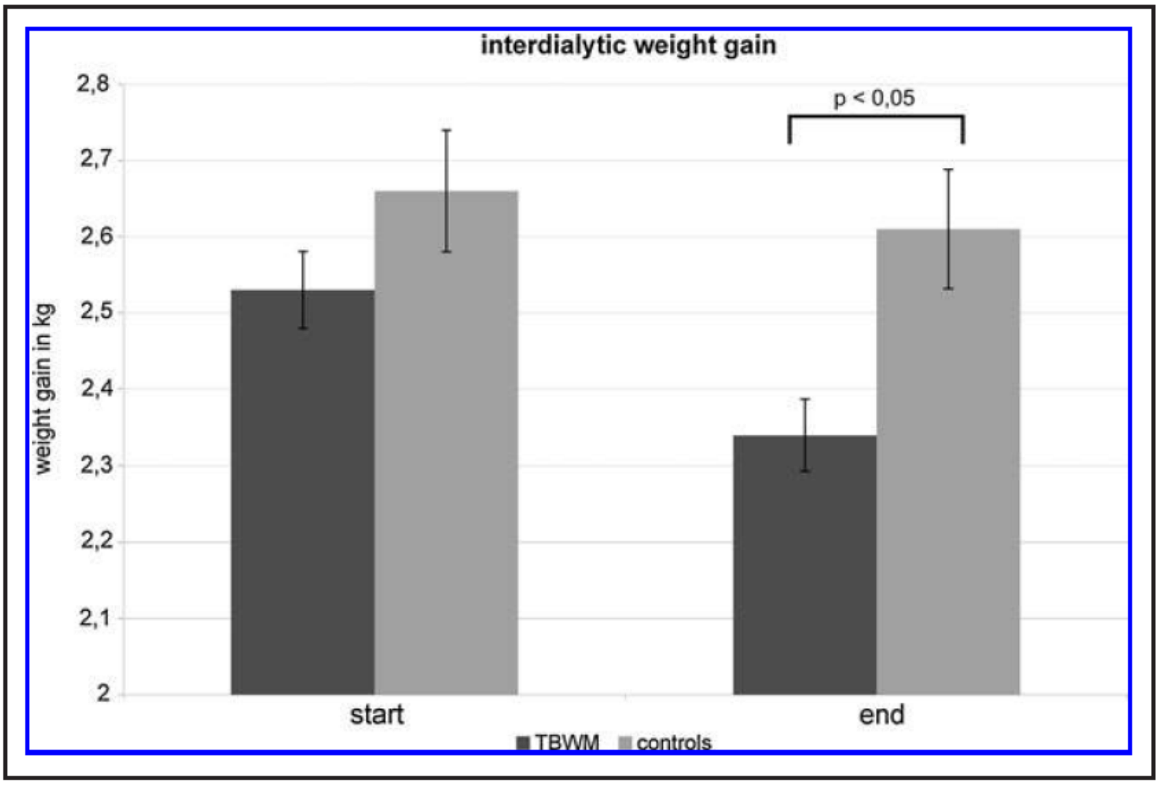

Fig. 3. Interdialytic weight gain at the end point (unfiltered). TBWM, telemetric body weight measurement.

[60] Sevick *et al.* (2005)

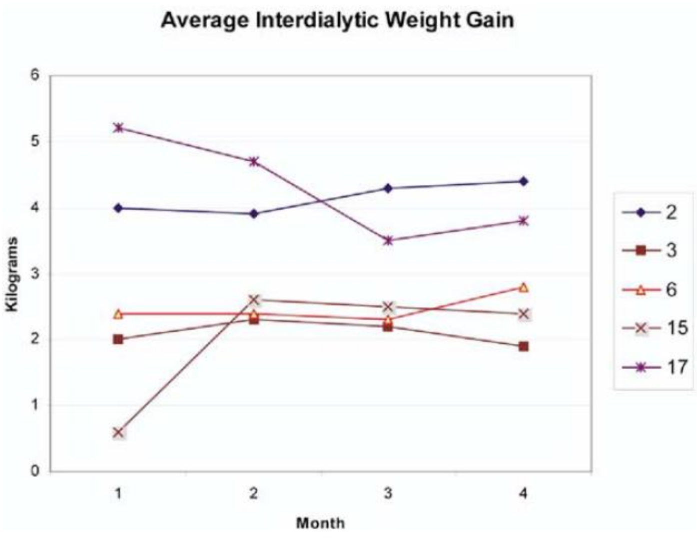

Figure 3. Monthly average interdialytic weight gain.

[69] Welch *et al*. (2013)

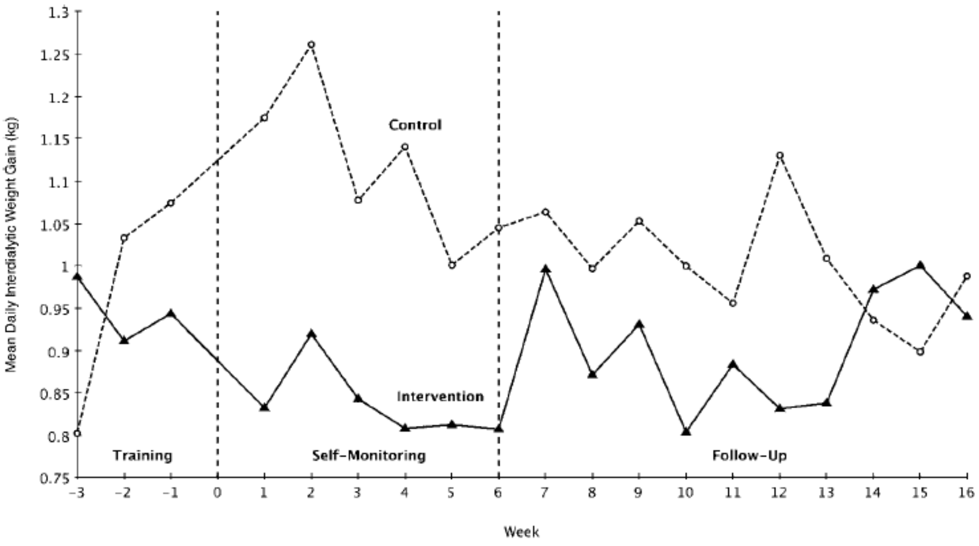

FIGURE 2. Mean daily interdialytic weight gain by week and group.

[76] Hayashi *et al*. (2017)

Multimedia Appendix 1 Table showing clinical parameters at baseline, study period and follow-up period in the Self-Management and Recording System for Dialysis group (n=9) and the non-Self-Management and Recording System for Dialysis group (n=11).

	Baseline	Study period	Follow-up period	
**SMART-D[a] group (n=9)**	mean (SD)			*P* value
IWG[b] (%DW[c]/day)	2.2 (0.4)	2.1 (0.3)	2.1 (0.4)	.68
Serum potassium concentrations (mEq/L)	4.9 (0.6)	5.1 (0.7)	5.0 (0.5)	.07
Serum phosphorus concentrations (mg/dL)	5.4 (1.2)	5.2 (1.0)	5.2 (1.2)	.67
**Non-SMART-D group (n=11)**	mean (SD)			*P* value
IWG (%DW/day)	2.2 (0.4)	2.3 (0.5)	2.4 (0.5)	.13
Serum potassium concentrations (mEq/L)	5.1 (0.8)	5.1 (0.6)	5.1 (0.7)	.84
Serum phosphorus concentrations (mg/dL)	5.0 (1.1)	5.0 (1.3)	5.2 (1.0)	.73

[a]SMART-D: Self-Management and Recording System for Dialysis.
[b]IWG: interdialysis weight gain.
[c]DW: dry weight.

Morbidity, mortality

[34] Ishani *et al.* (2016)

Table 2. Clinical End Points by Treatment Arm With Summary of Cox Proportional Hazards Analysis of Time-to-Event Variables

End Point	Intervention (n = 450)	Usual Care (n = 150)	HR (95% CI)
Primary	208 (46.2)	70 (46.7)	0.98 (0.75-1.29)
Secondary			
Death	13 (2.9)	3 (2.0)	1.46 (0.42-5.11)
Hospitalization	134 (29.8)	40 (26.7)	1.15 (0.80-1.63)
Emergency department visits	164 (36.4)	58 (38.7)	0.92 (0.68-1.24)
Admission to skilled nursing facility	18 (4.0)	2 (1.3)	3.07 (0.71-13.24)
Initiation of dialysis	11 (2.4)	2 (1.3)	1.86 (0.41-8.39)
Other[a]			
Second hospitalization[b]	46 (34.3)	16 (40.0)	0.89 (0.50-1.57)
Second emergency department visit[b]	64 (39.0)	26 (44.8)	0.87 (0.55-1.37)
No. of days of first hospital visit[c]	3.4 ± 3.5 [3]	3.6 ± 3.9 [2]	
No. of days of all hospitalizations[c]	5.7 ± 8.6 [3]	6.2 ± 6.5 [3]	
No. of hospitalizations within year[a]			
0	316 (70.2)	110 (73.3)	
1	88 (19.6)	24 (16.0)	
≥2	46 (10.2)	16 (10.7)	

Note: Values for categorical variables are given as number (percentage); for continuous variables, as mean ± standard deviation [median]. No significant differences were seen between groups in any of the measured outcomes.

Abbreviations: CI, confidence interval; HR, hazard ratio.

[a]Pearson χ^2 test for the categorized number of hospital admissions within the first year after randomization.

[b]Percentage of those with a first visit.

[c]Wilcoxon rank sum tests for duration of hospitalization.

[61] Gallar *et al.* (2007)

Table 2 Hospitalization rate in patients with and without telemedicine home support

Patients	Age (years) Mean (SD)	Charlson's co-morbidity index Mean (SD)	Hospitalization (days/patient/year) Mean (SD)
Group A – with telemedicine (*n*=25)	48 (10)	3.2 (1.4)	2.2 (2.4)
Group B – without telemedicine (*n*=32)	45 (16)	3.4 (1.9)	5.7 (9.0)
	P=0.403	*P*=0.580	*P*=0.043

Hospitalization rate, emergency room visits

[61] Gallar *et al.* (2007)

Table 2 Hospitalization rate in patients with and without telemedicine home support

Patients	Age (years) Mean (SD)	Charlson's co-morbidity index Mean (SD)	Hospitalization (days/patient/year) Mean (SD)
Group A – with telemedicine (*n*=25)	48 (10)	3.2 (1.4)	2.2 (2.4)
Group B – without telemedicine (*n*=32)	45 (16)	3.4 (1.9)	5.7 (9.0)
	P=0.403	*P*=0.580	*P*=0.043

[59] Berman *et al.* (2011) / [66] Minatodani *et al.* (2013)

Table 1. Healthcare Resource Outcomes

	REMOTE TECHNOLOGY *N*=19	USUAL CARE *N*=25	
	MEAN (SD)	MEAN (SD)	*P*-VALUE
Gender			
Females	7	11	
Males	12	14	
Total study days per patient	353 (208.39)	334 (202.63)	0.381
Range	108–649	138–714	
Age	56.78 (12.01)	62.00 (14.46)	0.190
Range	42–79	32–85	
Risk score	1.41 (0.16)	1.35 (0.11)	0.199
Range	1.217–1.827	1.200–1.510	
Charlson comorbidity index (modified)[a]	3.35 (1.90)	3.00 (1.82)	0.560
Range	0–6	0–7	
Karnofsky score	58.75 (3.15)	57.60 (5.22)	0.433
Range	50–60	40–60	
SF-36 (preintervention)	63.90 (15.50)	59.13 (16.85)	0.170
Range	34.4–88.0	21.7–90.0	
SF-36 (6–9 months)	60.76 (20.70)	59.50 (17.56)	0.417
Range	21–98	18–89	
Hospital days (per study day)	0.0082 (0.0225)	0.0355 (0.0555)	0.016[b]
Range	0–0.0926	0–0.1842	
Hospitalizations (per study day)	0.0018 (0.0029)	0.0056 (0.0062)	0.008[b]
Range	0–0.0093	0–0.0205	
ER visits (per study day)	0.0003 (0.0008)	0.0019 (0.0036)	0.035[b]
Range	0–0.0030	0–0.0132	
Hospital ER charges (per study day)	$114 (179.45)	$322 (336.53)	0.041[b]
Range	$6.99–$597.56	$0–$1,096.96	

[a]Hemmelgarn et al.[6]
[b]$p<0.05$.
ER, emergency room; SF-36, 36-Item Short Form Health Survey.

[34] Ishani *et al*. (2016)

Table 2. Clinical End Points by Treatment Arm With Summary of Cox Proportional Hazards Analysis of Time-to-Event Variables

End Point	Intervention (n = 450)	Usual Care (n = 150)	HR (95% CI)
Primary	208 (46.2)	70 (46.7)	0.98 (0.75-1.29)
Secondary			
Death	13 (2.9)	3 (2.0)	1.46 (0.42-5.11)
Hospitalization	134 (29.8)	40 (26.7)	1.15 (0.80-1.63)
Emergency department visits	164 (36.4)	58 (38.7)	0.92 (0.68-1.24)
Admission to skilled nursing facility	18 (4.0)	2 (1.3)	3.07 (0.71-13.24)
Initiation of dialysis	11 (2.4)	2 (1.3)	1.86 (0.41-8.39)
Other[a]			
Second hospitalization[b]	46 (34.3)	16 (40.0)	0.89 (0.50-1.57)
Second emergency department visit[b]	64 (39.0)	26 (44.8)	0.87 (0.55-1.37)
No. of days of first hospital visit[c]	3.4 ± 3.5 [3]	3.6 ± 3.9 [2]	
No. of days of all hospitalizations[c]	5.7 ± 8.6 [3]	6.2 ± 6.5 [3]	
No. of hospitalizations within year[a]			
0	316 (70.2)	110 (73.3)	
1	88 (19.6)	24 (16.0)	
≥2	46 (10.2)	16 (10.7)	

Note: Values for categorical variables are given as number (percentage); for continuous variables, as mean ± standard deviation [median]. No significant differences were seen between groups in any of the measured outcomes.

Abbreviations: CI, confidence interval; HR, hazard ratio.

[a]Pearson χ^2 test for the categorized number of hospital admissions within the first year after randomization.

[b]Percentage of those with a first visit.

[c]Wilcoxon rank sum tests for duration of hospitalization.

Medical cost

[59] Berman *et al.* (2011) / [66] Minatodani *et al.* (2013)

Table 1. Healthcare Resource Outcomes

	REMOTE TECHNOLOGY $N=19$	USUAL CARE $N=25$	
	MEAN (SD)	MEAN (SD)	P-VALUE
Gender			
Females	7	11	
Males	12	14	
Total study days per patient	353 (208.39)	334 (202.63)	0.381
Range	108–649	138–714	
Age	56.78 (12.01)	62.00 (14.46)	0.190
Range	42–79	32–85	
Risk score	1.41 (0.16)	1.35 (0.11)	0.199
Range	1.217–1.827	1.200–1.510	
Charlson comorbidity index (modified)[a]	3.35 (1.90)	3.00 (1.82)	0.560
Range	0–6	0–7	
Karnofsky score	58.75 (3.15)	57.60 (5.22)	0.433
Range	50–60	40–60	
SF-36 (preintervention)	63.90 (15.50)	59.13 (16.85)	0.170
Range	34.4–88.0	21.7–90.0	
SF-36 (6–9 months)	60.76 (20.70)	59.50 (17.56)	0.417
Range	21–98	18–89	
Hospital days (per study day)	0.0082 (0.0225)	0.0355 (0.0555)	0.016[b]
Range	0–0.0926	0–0.1842	
Hospitalizations (per study day)	0.0018 (0.0029)	0.0056 (0.0062)	0.008[b]
Range	0–0.0093	0–0.0205	
ER visits (per study day)	0.0003 (0.0008)	0.0019 (0.0036)	0.035[b]
Range	0–0.0030	0–0.0132	
Hospital ER charges (per study day)	$114 (179.45)	$322 (336.53)	0.041[b]
Range	$6.99–$597.56	$0–$1,096.96	

[a]Hemmelgarn et al.[6]
[b]$p<0.05$.
ER, emergency room; SF-36, 36-Item Short Form Health Survey.

[61] Gallar *et al.* (2007)

Table 1 Cost analysis for group A

	Hospital visits (€)	Teleconsultations (€)
Staff	23.45	13.88
Pharmaceuticals	58.92	58.92
Hospital space	19.45	19.45
Consumables (e.g. antiseptics, gauze, latex gloves, clamps)	15.25	
Videoconference equipment		55.18
Videoconference call costs		50.95
Transportation	60.00	
Total	*177.07*	*198.40*

Cost effectiveness

[70] Blakeman *et al.* (2014)

Based on multiple imputation data, the intervention group was associated with a reduction in costs compared with control due to lower secondary care attendance. The mean difference in total cost was £175 (95%CI 2£284 to £635). Control groups costs were inflated by two individuals spending 60+ days as inpatients. However, even when these are removed, the intervention is associated with reduced costs (mean difference £123, 95% CI 2 £103 to £349). Results from the multiple imputation were consistent with the complete case analysis, where again QALYs were significantly higher in BRIGHT group and costs were reduced.

Nutrition, dietary intake

[62] Whitten *et al.* (2008)

Table 3 Comparisons of clinical data to Renal Network 11 recommendations and best practices

	Renal Network 11 recommendation	Renal Network 11 best practices	Six-month average
Haemoglobin	≥80% of patients should have haemoglobin ≥11 g/dL	>85% of patients have haemoglobin ≥11 g/dL	89%*†
Urea reduction ratio	≥80% of patients should have urea reduction ratio ≥65%	>85% of patients will have urea reduction ratio ≥65%	98%*†
Albumin	≥80% of patients should have mean serum albumin ≥ lower limit of normal (3.4 g/dL)	>85% of patients have a mean serum albumin ≥ lower limit of normal (3.4 g/dL)	76%
	≤10% of patients should have mean serum album <0.9 lower limit of normal (3.1 g/dL)	≤10% of patients with a mean serum album <0.9 lower limit of normal (3.1 g/dL)	7%*†
Phosphorus	≥70% of patients should have mean serum phosphorus ≤5.5 mg/dL	>80% of patients should have mean serum phosphorus ≤5.5 mg/dL	74%*
	<10% of patients should have mean serum phosphorus >8 mg/dL	<5% of patients should have mean serum phosphorus >8 mg/dL	0%*†
Calcium	≥80% of patients should have mean corrected serum calcium concentration ≤10.2 mg/dL	>85% of patients should have mean corrected serum calcium concentration ≤10.2 mg/dL	91%*†
		>75% of patients should have a mean corrected calcium concentration ≤9.5 mg/dL	33%

*Clinical data from investigation met Renal Network 11 recommendations
†Clinical data from investigation met Renal Network 11 recommendations for best practices

[69] Welch *et al.* (2013)

Table 2. Comparison of Mean Fluid and Dietary Intake in the Dietary Intake Monitoring Application (DIMA) Group at Baseline and End of Self-Monitoring

	All DIMA Subjects (n = 14)					DIMA Active Users Only (n = 11)				
	Week 1		Week 6			Week 1		Week 6		
	M	*SD*	*M*	*SD*	*p*	*M*	*SD*	*M*	*SD*	*p*
Fluid, ml	503.4	340.6	469.0	297.7	.76	559.9	363.8	466.1	209.7	.45
Sodium, mg	2381.7	1751.7	1498.2	738.6	.12	2778.7	1759.5	1434.2	618.0	.05
Potassium, mg	1592.6	1251.0	1060.2	757.5	.16	1758.4	1322.0	989.0	542.5	.10
Phosphorus, mg	872.8	785.7	568.1	393.3	.21	991.1	830.4	515.5	193.7	.11
Protein, g	59.7	42.1	40.6	22.7	.18	68.0	43.3	38.2	13.2	.08
Calories, kcal	1335.6	862.1	835.7	495.8	.09	1496.1	887.6	777.2	273.1	.04

Note: DIMA active users were defined as those who used DIMA at least 50% of the intervention period.

Medication adherence

[35] Reese *et al.* (2017)

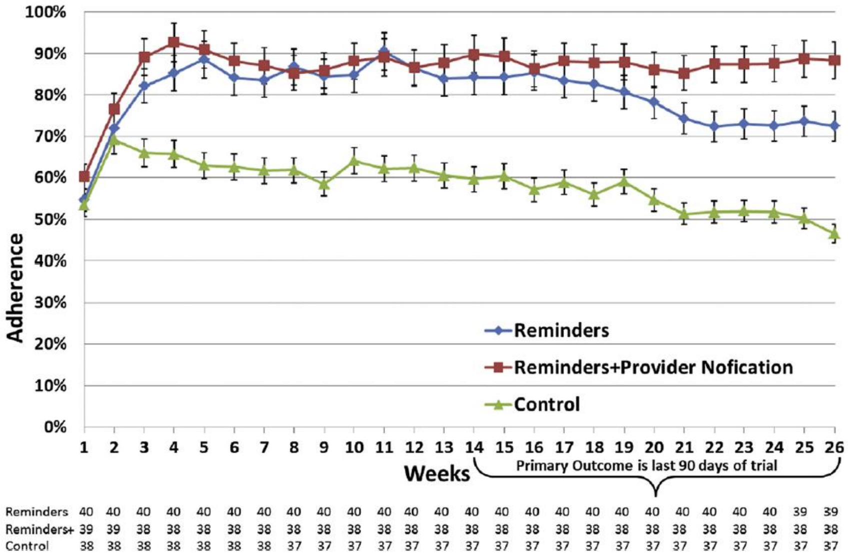

Figure 2. Weekly proportion of participants with pill bottle opening.

[36] McGillicuddy *et al.* (2013)

Table 2. Medication adherence by time across treatment condition (Bonferroni adjusted 95% confidence intervals[CI]).

Medication Adherence by Time Across Treatment Condition	mHealth (n=9)			Standard Care (n=10)		
	Mean	SE	CI (95%)	Mean	SE	CI (95%)
Baseline	.576	.048	.474-.677	.500	.046	.404-.597
Month 1	.87.4	.046	.777-.970	.533	.043	.442-.625
Month 2	.929	.040	.844-1.014	.587.	038	.507-.668
Month 3	.945	.037	.865-1.025	.574	.036	.498-.650

[66] Forni Ogna *et al.* (2013)

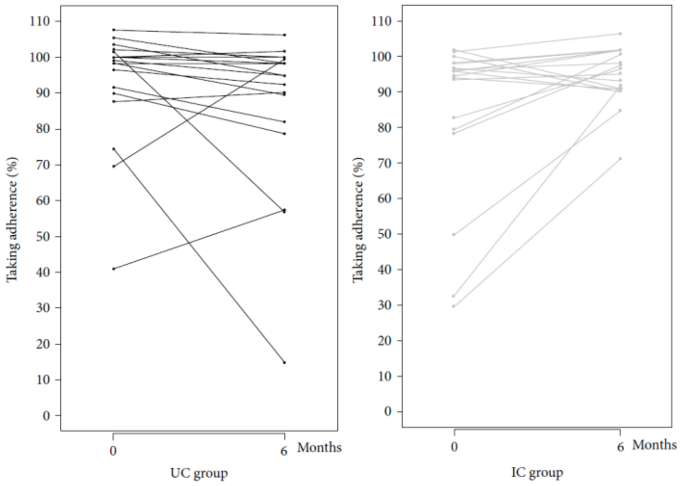

* *P* = 0.02 (Wilcoxon rank-sum test)

FIGURE 5: Change in individual taking adherence over six-month MEMS monitoring.

[33] Rifkin *et al.* (2013)

Table 3 **Comparison of telemonitoring and usual care groups at start and conclusion**

	Telemonitoring	Usual care	*P* for two-group comparison
Systolic blood pressure (mmHg)			
Start of study	147±17.5	146±8.8	0.87
End of study	136±15.6	140±14.4	0.48
Change	−13 (0, −31); *P*=0.009 vs. start	−8.5 (0, −18); *P*=0.031 vs. start	0.32
Diastolic blood pressure (mmHg)			
Start of study	77±12.3	81±10.9	0.35
End of study	73±10.3	73±12.6	0.93
Change	−3.5 (−3, 12.5); *P*=0.02 vs. start	−8 (3, 13); *P*=0.0079 vs. start	0.35
Mean arterial pressure (mmHg)			
Start of study	101±11	102.9±9.6	0.46
End of study	93.9±8.6	95.2±11.7	0.67
Change	−7.13 (−16.5, 0.05)	−7.67 (−12.3, −1.7)	0.87
Creatinine (mg/dl)			
Start of study	2.13±0.66	1.96±0.53	0.42
End of study	2.17±0.76	2.32±0.84	0.64
Change	0.04 (−0.09, 0.34)	0.28 (0, 0.34)	0.12
eGFR (ml/min/1.73 m^2)			
Start of study	37.3±14.2	39.4±10.6	0.64
End of study	37.9±16.7	34.5±13.2	0.51
Change	0.6 (−3.4, 1.8)	−3.69 (−6.2, 0)	0.14
Total number of medications			
Start of study	11.2±4.1	11.1±5.6	0.91
End of study	12±4.6	12.8±5.1	0.62
Change	0.96 (0, 2)	1.53 (0, 3)	0.33
Number of blood pressure medications			
Start of study	3.9±1.4	3.9±1.4	0.92
End of study	4±1.2	3.9±1.3	0.61
Change	0.89 (−1, 4)	1 (0, 2)	0.91
Total number of interventions	4.6±2.1	1.7±0.97	<0.01
Morisky Medication Adherence Scale			
Start of study	6.9±1.3	6.4±1.7	0.35
End of study	7±1.2	7.2±1.4	0.58
Change	0.11 (0, 0.5)	0.67 (0, 2)	0.17

Data are presented as means±SD, median and IQR, or percentages.
Morisky Medication Adherence Scale, 8 represents perfect adherence, 6–7 represent medium adherence, 5 and below represent poor adherence.
eGFR, estimated glomerular filtration rate.

Acceptability

[69] Welch *et al.*(2013)

Results: On the scale of 1–5, the mean acceptability score in the DIMA group was 3.93 (SD ¼ 0.43, median ¼ 3.86, range 2.68–4.68). Items ranked more favorably were (a) satisfied using the monitor for my food and diet intake (M ¼ 4.33), (b) easy to use (M ¼ 4.28), and (c) feedback was helpful (M ¼ 4.28). Items ranked less favorably included (a) needed more information than provided (M ¼ 3.33), (b) the scanner was helpful (M ¼ 3.44), and (c) easily found find the icons needed (M ¼ 3.61). During the self-monitoring period, DIMA use ranged from 2 to 48 days. The monitoring period went beyond the scheduled 42 days in some participants due to dialysis scheduling, an illness, or PDA problems. Five participants (26.4%) used the application on less than half the number of days available, and five (26.4%) used it more than 80% of the time.

[74] Dey *et al.* (2016)

Table 3. QUEST and QOL scores at the start and end of programme.

	Initial	Final	p-Values
QUEST,[a] mean (SD)			
Device	4.5 (0.5)	4.5 (0.6)	
Service	4.2 (0.9)	4.1 (1.0)	
Total (device + service)	4.4 (0.5)	4.3 (0.6)	
QOL, mean (SD)[b]			
Symptom/problem list	70.2 (17.0)	64.7 (15.8)	0.27
Effects of kidney disease	75.6 (21.5)	69.6 (24.3)	0.37
Burden of kidney disease	55.1 (33.9)	40.9 (24.7)	0.49
SF-12 Physical Health Composite	29.7 (6.1)	31.5 (8.8)	0.54
SF-12 Mental Health Composite	46.2 (10.4)	43.6 (11.6)	0.43

QUEST: Quebec User Evaluation of Satisfaction with assistive Technology; SD: standard deviation; QOL: quality of life; SF-12: short form-12.
[a]Scores on a scale of 1–5 with 1 indicating *not satisfied at all* and 5 being *very satisfied*.
[b]Scores on a scale of 0–100 with higher scores indicating better quality of life.

[76] Hayashi *et al.* (2017)

All 9 patients in the SMART-D group were able to complete the 2-week use of the system without any major problems. The mean number of daily entries for the dialysis date when there were 4 items to be entered (body weight in the morning and afternoon, predialysis weight, and postdialysis weight) was 3.9 (SD 0.2). The mean number of daily entries for the nondialysis date when there were 2 items to be entered (body weight in the morning and afternoon) was 1.8 (SD 0.5). The average completion rates for body weight in the morning and the afternoon and predialysis/postdialysis weight were, respectively, 89% (SD 23) and 95% (SD 7). The average completion rate for serum potassium and phosphorus concentrations was 78% (SD 44).

Table 2. Average number of daily entries and completion rates (n=9).

	Mean (SD)
**Mean number of daily entries**	
Dialysis date (4 items to be entered)	3.9 (0.2)
Nondialysis date (2 items to be entered)	1.8 (0.5)
**Completion rate (%)**	
Body weight in the morning and the afternoon	89 (23)
Predialysis/postdialysis weight	95 (7)
Serum potassium and phosphorus concentrations	78 (44)

[33] Rifkin *et al.* (2013)

Overall, satisfaction with the device was high among participants with 96% reporting that they would continue using the device (Box 1). Similarly, clinic physicians also rated the device as a highly acceptable intervention, with 7/7 regular clinic physicians surveyed stating that they felt the patients benefited from the system in terms of BP control.

**Box 1: Participant feedback**
The whole thing is so simple and easy. If I have any blood pressure problems, the doctor knows right away. I'm sad that I don't get to keep using it.<br>It made me check my blood pressure more because I knew people were looking at the readings.<br>Really liked it. It was easy for me to hook it up and keep track.<br>It's like special treatment because the doctor was able to call and fix my medications.

[36] McGillicuddy *et al.* (2013)

Results: The mHealth group reported high overall satisfaction with the mHealth system (average score 4.8/5 point Likert scale: 1= strongly disagree-5 = strongly agree). The mHealth system was easy for the subjects to learn to use (4.7/5) and easy to use in their home (4.8/5). They also found the system useful for medication and health management (4.3/5). Physicians of the mHealth subjects received weekly reports via email detailing their patients' adherence rates and average blood pressures. Armed with the information provided, physicians of mHealth patients prescribed more medication changes to anti-hypertensive medications (7 changes in 5 patients) than controls (3 changes in 3 patients).

[75] Ong *et al.* (2016)

The acceptability of the smartphone self–management application was assessed by determining its adoption, adherence to recommended scheduled use, user satisfaction (interview), and feature usage. Integrating a smartphone–based self–management systemin to usual care of patients with advanced CKD proved feasible and acceptable, and it appeared to be clinically useful.

Usability

[64] Connelly *et al.* (2012)

A 27-item post-intervention usability questionnaire developed by Susan Rawl and colleagues, modified for DIMA

A 33-item post-intervention questionnaire measuring the usability of specific features and context of use, developed specifically for the DIMA pilot. Subjects were able to use DIMA successfully-12 subjects used DIMA as much or more at the end of the study as they did at the beginning and reported that DIMA helped them change their diet.

[67] Heiden *et al.* (2013)

The usability testing and the qualitative interviews mainly resulted in positive feedback from the participants. Most of the functions and content of the system were considered relevant and useful according to daily challenges and experienced scenarios.

[76] Hayashi *et al.* (2017)

Of the 7 participants who answered an end-of-study usability survey, all were motivated by the sense of security derived from using the system.

Table 3. Usability survey results.

Statement or question (n=7)	Response
I could use a smartphone with no problem. (n [%])	5 (71)
I could use a weight scale with no problem. (n [%])	6 (86)
The interface of the data registration was easy to use. (n [%])	7 (100)
The interface of the data browsing was easy to watch. (n [%])	6 (86)
The instructions were easy to understand. (n=5[a]) (n [%])	5 (100)
The devices caused me physical discomfort. (n [%])	0 (0)
I easily incorporated using the system into my daily routine. (n [%])	5 (71)
Using the system gave me a sense of security. (n [%])	7 (100)
Participation in the study helped me to improve lifestyle and dialysis self-management. (n [%])	6 (86)
Using the system caused me some problems. (n [%])	1(14)[b]
How much time did you spend using the system per day? (minutes, mean [SD])	7.7 (3.9)
Is the system worth using for the time you spent? (n [%])	6 (86)
Did this system give a positive impact on your dialysis management? (n [%])	6 (86)
Would you like to continue using this system? (n [%])	5 (71)[c]

[a]Two participants answered that they did not use the instructions.
[b]One participant felt that using the SMART-D was time-consuming.
[c]The reasons given by the participants are shown in Table 4.

[62] Whitten *et al.* (2008)

Table 2 Provider perceptions of telehealth consultations (scored from 0 = strongly disagree to 7 = strongly agree)

	Mean	SD
I found the telehealth service easy to use	7.0	0.0
I think that telehealth is a good way to provide medical care	5.5	1.7
It was easy to communicate with the other person during the telehealth consultation	6.0	0.8
I enjoy telehealth visits	5.8	1.0
The patient felt comfortable with the telehealth equipment	5.5	0.6
The patient was able to communicate what was bothering him/her	6.0	0.8
I felt comfortable using the telehealth equipment	7.0	0.0
The care that I provided via the telehealth consultation was as good as a regular in-person visit	5.8	0.5
Overall, I am satisfied with the telehealth service that I provided	6.5	0.6
I would rather see the patient in person than via the telehealth equipment	4.0	2.4
The telehealth equipment increases my access to my patients	7.0	0.0
I felt that telehealth limited my patient's privacy	4.5	1.0
Telehealth should only be used when a health-care professional cannot be physically present	3.0	2.2
More people should have access to telehealth programmes	6.8	0.5

Table 1 Patient perceptions of telehealth consultations (scored from 0 = strongly disagree to 7 = strongly agree)

	Mean	SD
I found the telehealth service easy to use	6.1	1.2
I think that telehealth is a good way to provide medical care	5.8	1.5
It was easy to communicate with the other person during the telehealth consultation	6.3	1.0
I enjoy telehealth visits	5.7	1.4
I felt comfortable using the telehealth equipment	6.1	1.4
I was able to communicate what was bothering me	6.0	1.5
The health-care provider was able to address what was bothering me	6.1	1.6
The care that I received via the telehealth consultation was as good as an ordinary in-person visit	5.2	1.8
Overall, I am satisfied with the telehealth service that I received	6.2	1.1
I would rather be seen in person than via the telehealth equipment	4.8	2.0
I believe that the telehealth equipment increases my access to health care	5.8	1.3
I felt that telehealth limited my privacy	3.2	2.0
Telehealth should only be used when a health-care professional cannot be physically present	4.7	2.3
More people should have access to telehealth programmes	6.0	1.2

[77] Liu *et al.* (2017)

Results: Patients reported an average of 4.2 on ease of use of the system (SD 0.77). Patients also reported an average of 4.1 for reliability and performance of the system (SD 0.87). They also gave positive feedback regarding the usage of the system during their interviews. The nurses reported satisfaction with the system features during the interview.

Satisfaction

[36] McGillicuddy *et al.* (2013)

The mHealth group reported high overall satisfaction with the mHealth system (average score 4.8/5 point Likert scale: 1= strongly disagree-5 = strongly agree). The mHealth system was easy for the subjects to learn to use (4.7/5) and easy to use in their home (4.8/5). They also found the system useful for medication and health management (4.3/5).

[59] Berman *et al.* (2011) / [58] Minatodani *et al.* (2013)

Overall, patients reported high levels of satisfaction with RCN support as they received efficient feedback, were better able to identify changes in their health status, and experienced enhanced accountability, self-efficacy, and motivation to make health behavior changes.

[71] Harrington *et al.* (2014)

Table 3. Summary of the Likert evaluation of iPad application

Question	Mean	Mini-mum	Maxi-mum
I used the step-by-step instructions each time I did an exchange	6.0	0	10
The step-by-step instructions were helpful for keeping sterility	7.0	0	10
I recorded my exchanges as I was performing them	5.6	0	10
I prefer to use PD Remote for keeping track of my exchanges	6.8	4	10
I feel PD Remote helped me stay on schedule for my exchanges better than the paper record	1.6	0	4
Using the camera feature was helpful for recording my exit site problems	5.3	0	10
Using the camera feature was helpful for recording my edema	3.3	0	10
Using the camera feature was helpful for recording my PD fluid characteristics	3.3	0	10
Using the video feature was helpful for recording my exit site problems	3.3	0	10
Using the video feature was helpful for recording my edema	3.3	0	10
Using the video feature was helpful for recording my PD fluid characteristics	4.0	0	10
I felt like I had more security with doing exchanges since my records were able to be viewed by the clinic	6.8	2	10
The laboratory data was able to be viewed without any problems	2.2	0	6
There were no issues with the connection during my use with PD Remote	2.4	0	8
I would prefer to continue using PD Remote rather than paper charting	4.2	0	10
My overall experience with PD Remote was great and I would prefer to use it over paper recording	5.2	0	9

[72] Diamantidis *et al.* (2015)

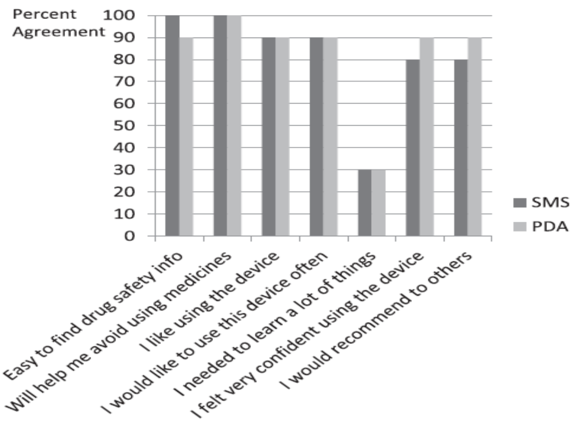

Figure 2. | **Medication inquiry system satisfaction survey by device type.** Percentage of participants in agreement with satisfaction survey questions. PDA, personal digital assistant; SMS, short messaging service.

[73] van Lint *et al.* (2015)

Table 4 Experience of advantages and disadvantages of self-monitoring at baseline and follow-up

	Baseline		Follow-up		Dependent t-test
	**Mean***	**SD**	**Mean***	**SD**	***P*-value**
It is an advantage that ...					
I can control creatinine regularly	4.20	0.91	3.84	1.38	0.15
I can control blood pressure regularly	4.20	0.91	4.08	1.15	0.60
Physicians have direct access to results	4.72	0.46	3.96	1.31	<0.01
Changes in my condition are noticed quickly	4.76	0.52	4.08	1.19	0.02
I have to take up a more active role in my own care	4.68	0.56	4.04	1.06	<0.01
I receive a warning in case of raised creatinine levels	4.76	0.44	4.20	1.04	0.01
It is a disadvantage that ...					
I have to perform measurements myself	1.60	1.00	1.36	0.91	0.16
Performing the measurements takes time	2.00	1.32	1.48	0.92	0.05
I have to interpret results myself	1.72	1.17	1.16	0.47	0.03
I have to keep track of values digitally	1.88	1.13	1.12	0.44	<0.01
I have to take up a more active role in my own care	1.68	1.18	1.36	0.91	0.12

Note: *On a scale of 1 (disagree) to 5 (agree).
Abbreviation: SD, standard deviation.

[74] Dey *et al.* (2016)

Table 3. QUEST and QOL scores at the start and end of programme.

	Initial	Final	p-Values
QUEST,[a] mean (SD)			
Device	4.5 (0.5)	4.5 (0.6)	
Service	4.2 (0.9)	4.1 (1.0)	
Total (device + service)	4.4 (0.5)	4.3 (0.6)	
QOL, mean (SD)[b]			
Symptom/problem list	70.2 (17.0)	64.7 (15.8)	0.27
Effects of kidney disease	75.6 (21.5)	69.6 (24.3)	0.37
Burden of kidney disease	55.1 (33.9)	40.9 (24.7)	0.49
SF-12 Physical Health Composite	29.7 (6.1)	31.5 (8.8)	0.54
SF-12 Mental Health Composite	46.2 (10.4)	43.6 (11.6)	0.43

QUEST: Quebec User Evaluation of Satisfaction with assistive Technology; SD: standard deviation; QOL: quality of life; SF-12: short form-12.
[a]Scores on a scale of 1–5 with 1 indicating *not satisfied at all* and 5 being *very satisfied.*
[b]Scores on a scale of 0–100 with higher scores indicating better quality of life.

[76] Hayashi *et al.* (2017)

Results: A total of 7 of them completed questionnaires rating their assessment of SMART-D's usability and their satisfaction with the system. 6 of the 7 (86%) reported that using SMART-D helped improve their lifestyle and self-management.

[75] Ong *et al.* (2016)

Results: Of 47 patients, 38 patients participated in an exit interview; four declined, three had severe medical complications or were hospitalized at the time of interviews, and two had died. All but two indicated that the application made them feel more connected with their health care providers and that they wished to continue using it after the study The nurses found that the system helped them prioritize the patients who needed more attention. The pharmacists felt that the medication review alerts lead to interventions that mitigated adverse drug events. None reported disruption in their workflow, and all endorsed future use of the smartphone system.

Adherence to intervention

[35] Reese *et al.* (2017)

Table 3. Self-reported Adherence Using BAASIS

Variables	Total (N = 117)	Reminders (n = 40)	Reminders + Notification (n = 39)	Control (n = 38)	Test Statistic; *P*
Adherent					0.58 (χ); 0.8
No	26 (22)	9 (23)	10 (26)	7 (18)	
Yes	91 (78)	31 (78)	29 (74)	31 (82)	
What percentage do you feel you took your tacrolimus on time?					2.18 (K); 0.3
Median	100 [95-100]	100 [95-100]	100 [95-100]	99 [95-100]	
No. of missing	12	4	4	4	

Note: Unless otherwise indicated, values are given as number (percentage) or median [interquartile range].
Abbreviations: BAASIS, Basel Assessment of Adherence to Immunosuppressive Medications Scale; χ, Pearson χ^2 test; K, Kruskal-Wallis test.

[63] Stark *et al.* (2011)

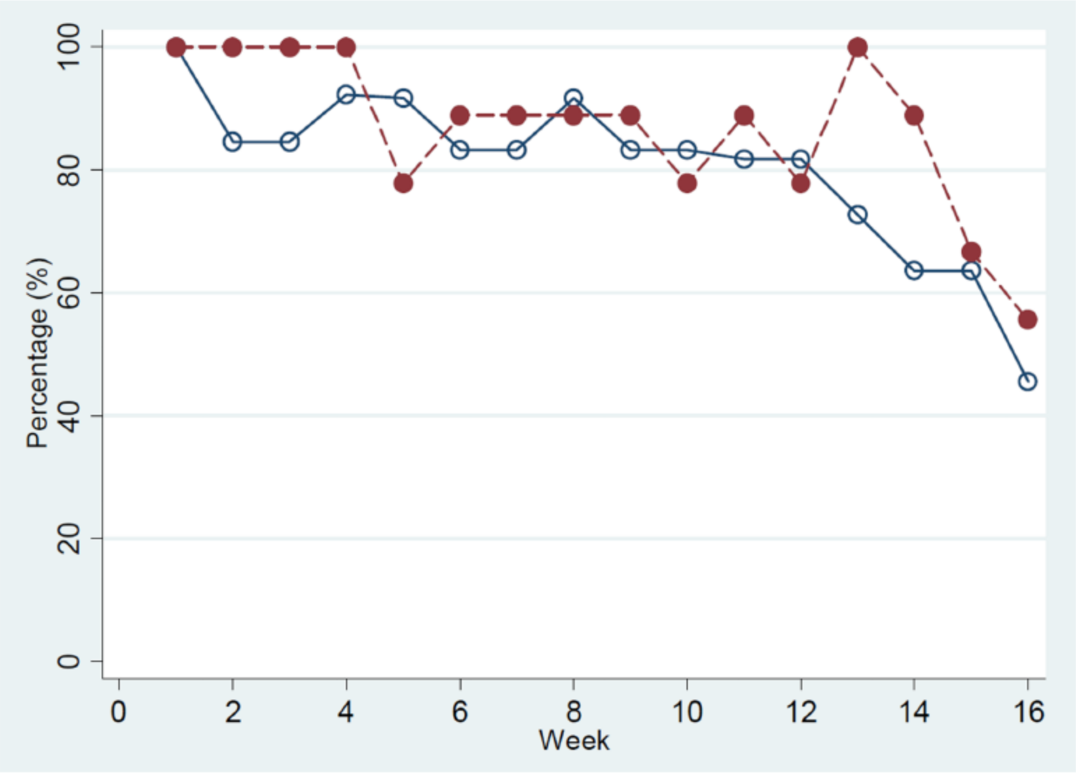

Figure 3.
Weekly percentage of participants who enter any meals in the BalanceWise-HD and BalanceWise-PD Studies

[73] van Lint *et al.* (2015)

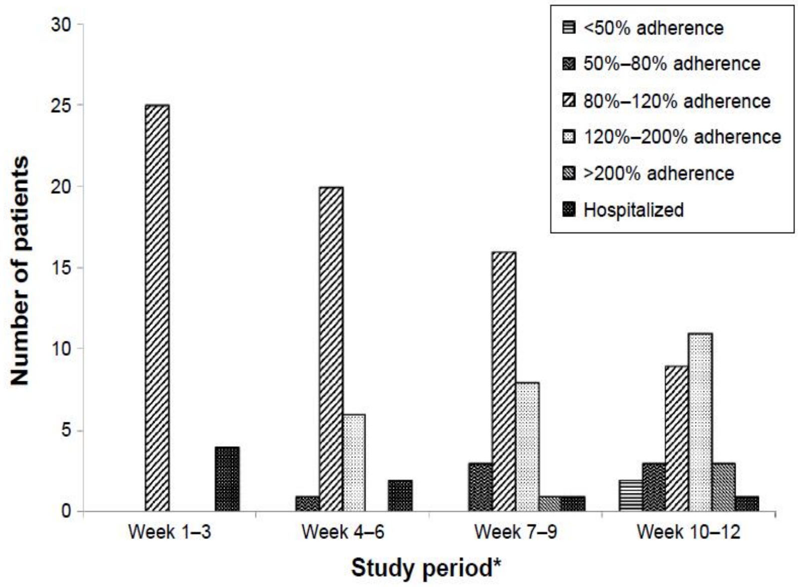

Figure 2 Percentage adherence to measurement frequency per protocol.
Notes: *Measurement frequency: week 1–3, daily; week 4–6, every other day; week 7–9, twice a week; week 10–12, weekly.

[75] Ong *et al.* (2016)

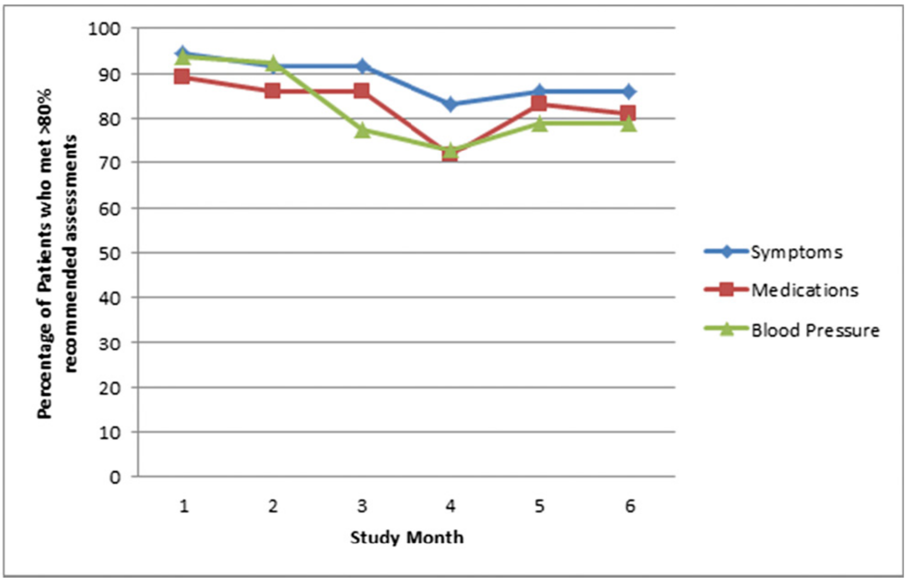

Figure 2. | **Adherence rates per month (symptoms, medications, and BP assessments using the mobile application).**

First entry, length of dwell time

[65] Diamantidis *et al*. (2013)

Figure 2. Cumulative incidence of Safe Kidney Care website log-in.

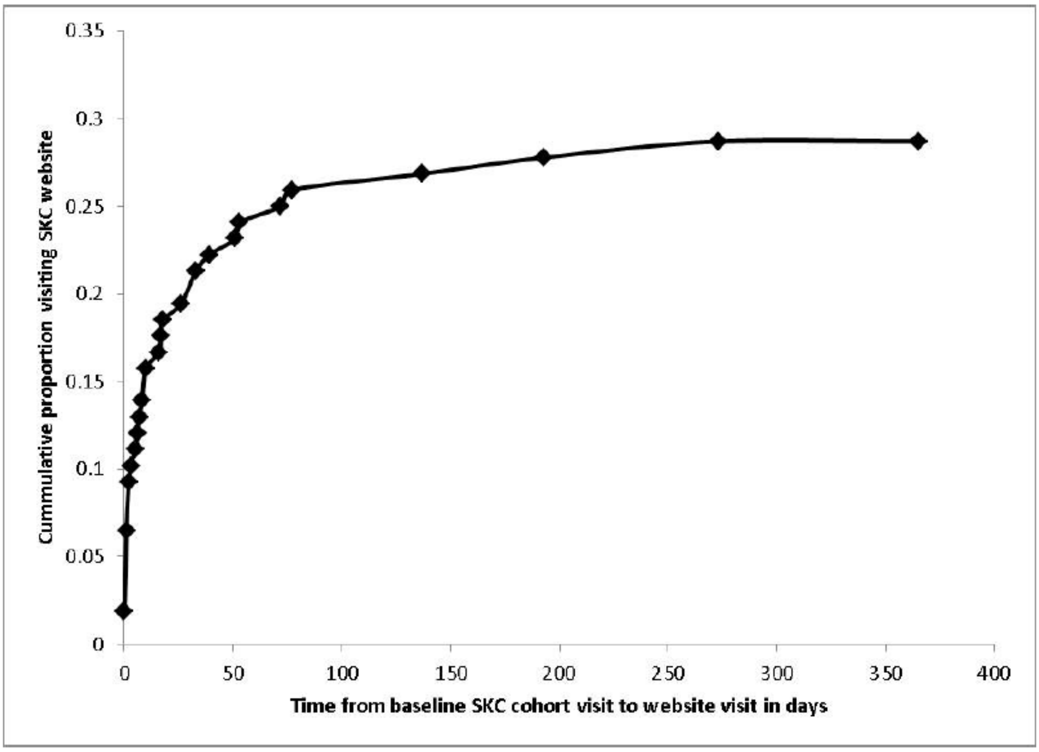

Self-efficacy

[69] Welch *et al*. (2013)

Table 3. Change Over Time and Treatment Effect on Self-Efficacy, Perceived Benefits, and Perceived Control

	Baseline			End of Self-Monitoring			Eight-Week Follow-Up			Treatment-by-Time Interaction	Treatment Effect[a]
	n	M	SD	n	M	SD	n	M	SD	p	p
Diet self-efficacy											
Intervention	23	35.5	9.5	18	32.7	10.1	16	33.2	12.4	.52	.40
Control	19	34.2	10.0	17	31.1	10.2	17	28.8	6.8		
Fluid self-efficacy											
Intervention	23	40.7	5.6	18	41.4	5.8	16	43.6	5.7	.39	.21
Control	19	41.0	7.9	18	43.9	6.4	17	46.6	5.4		
Perceived benefits of sodium adherence											
Intervention	23	30.3	3.0	18	29.9	4.4	16	31.6	2.8	.59	.77
Control	20	30.7	4.0	18	30.3	4.2	17	31.0	3.8		
Perceived benefits of fluid adherence											
Intervention	22	37.3	4.7	18	37.5	4.9	16	39.8	4.5	.48	.28
Control	20	38.5	4.6	18	39.6	6.1	17	40.1	4.9		
Perceived control											
Intervention	23	26.5	6.1	18	28.5	4.9	16	26.6	5.2	.01	—
Control	20	25.4	4.6	17	23.6	4.3	17	25.1	5.2		

[a]When no treatment by time interaction is present ($p > .1$).

Perceived benefits

[69] Welch *et al*. (2013)

Table 3. Change Over Time and Treatment Effect on Self-Efficacy, Perceived Benefits, and Perceived Control

	Baseline			End of Self-Monitoring			Eight-Week Follow-Up			Treatment-by-Time Interaction	Treatment Effect[a]
	n	M	SD	n	M	SD	n	M	SD	p	p
Diet self-efficacy											
Intervention	23	35.5	9.5	18	32.7	10.1	16	33.2	12.4	.52	.40
Control	19	34.2	10.0	17	31.1	10.2	17	28.8	6.8		
Fluid self-efficacy											
Intervention	23	40.7	5.6	18	41.4	5.8	16	43.6	5.7	.39	.21
Control	19	41.0	7.9	18	43.9	6.4	17	46.6	5.4		
Perceived benefits of sodium adherence											
Intervention	23	30.3	3.0	18	29.9	4.4	16	31.6	2.8	.59	.77
Control	20	30.7	4.0	18	30.3	4.2	17	31.0	3.8		
Perceived benefits of fluid adherence											
Intervention	22	37.3	4.7	18	37.5	4.9	16	39.8	4.5	.48	.28
Control	20	38.5	4.6	18	39.6	6.1	17	40.1	4.9		
Perceived control											
Intervention	23	26.5	6.1	18	28.5	4.9	16	26.6	5.2	.01	—
Control	20	25.4	4.6	17	23.6	4.3	17	25.1	5.2		

[a]When no treatment by time interaction is present ($p > .1$).

Perceived control

[69] Welch *et al*. (2013)

Table 3. Change Over Time and Treatment Effect on Self-Efficacy, Perceived Benefits, and Perceived Control

	Baseline			End of Self-Monitoring			Eight-Week Follow-Up			Treatment-by-Time Interaction	Treatment Effect[a]
	n	M	SD	n	M	SD	n	M	SD	p	p
Diet self-efficacy											
Intervention	23	35.5	9.5	18	32.7	10.1	16	33.2	12.4	.52	.40
Control	19	34.2	10.0	17	31.1	10.2	17	28.8	6.8		
Fluid self-efficacy											
Intervention	23	40.7	5.6	18	41.4	5.8	16	43.6	5.7	.39	.21
Control	19	41.0	7.9	18	43.9	6.4	17	46.6	5.4		
Perceived benefits of sodium adherence											
Intervention	23	30.3	3.0	18	29.9	4.4	16	31.6	2.8	.59	.77
Control	20	30.7	4.0	18	30.3	4.2	17	31.0	3.8		
Perceived benefits of fluid adherence											
Intervention	22	37.3	4.7	18	37.5	4.9	16	39.8	4.5	.48	.28
Control	20	38.5	4.6	18	39.6	6.1	17	40.1	4.9		
Perceived control											
Intervention	23	26.5	6.1	18	28.5	4.9	16	26.6	5.2	.01	—
Control	20	25.4	4.6	17	23.6	4.3	17	25.1	5.2		

[a]When no treatment by time interaction is present ($p > .1$).

Recorded errors

[72] Diamantidis *et al*. (2015)

Of 60 total medication queries, there were only three recorded errors, two of which occurred in the short messaging service texting group.
