# Supplementary material for: Electronic Health Self-Management Interventions for Patients With Chronic Kidney Disease: Systematic Review of Quantitative and Qualitative Evidence
Source: J Med Internet Res. 2019 Nov 5;21(11):e12384. doi: 10.2196/12384 (PMC6864489; doi:10.2196/12384)
Supplement: Multimedia Appendix 6 [file jmir_v21i11e12384_app6.pdf]

Multimedia Appendix 6 Most frequently recommended electronic health self-management intervention components

- Self-monitoring [60, 63, 73, 76]
- Message/alert to patients from health caregivers [35, 59, 71, 74]
- Counselling [33, 59, 74]
- Educational material or training on how to use eHealth [70, 72]
- Message/alert to health caregivers [74, 75]
- Message/alerts to patients from device [35, 75]
- Interactive/immediate feedback from the device [64]
